# Regulation of pattern recognition receptor signaling by palmitoylation

**DOI:** 10.1016/j.isci.2024.111667

**Published:** 2024-12-20

**Authors:** Xiaocui Li, Xiaofang Hu, Hongjuan You, Kuiyang Zheng, Renxian Tang, Fanyun Kong

**Affiliations:** 1 Jiangsu Key Laboratory of Immunity and Metabolism, Department of Pathogenic Biology and Immunology, Xuzhou Medical University, Xuzhou, Jiangsu, China; 2 Department of Breast Surgery, The Second Affiliated Hospital of Shandong First Medical University, Taian, Shandong, China; 3 National Demonstration Center for Experimental Basic Medical Sciences Education, Xuzhou Medical University, Xuzhou, Jiangsu, China

**Keywords:** Biological sciences, Molecular biology, Immune response

## Abstract

Pattern recognition receptors (PRRs), consisting of Toll-like receptors, RIG-I-like receptors, cytosolic DNA sensors, and NOD-like receptors, sense exogenous pathogenic molecules and endogenous damage signals to maintain physiological homeostasis. Upon activation, PRRs stimulate the sensitization of nuclear factor κB, mitogen-activated protein kinase, TANK-binding kinase 1-interferon (IFN) regulatory factor, and inflammasome signaling pathways to produce inflammatory factors and IFNs to activate Janus kinase/signal transducer and activator of transcription signaling pathways, resulting in anti-infection, antitumor, and other specific immune responses. Palmitoylation is a crucial type of post-translational modification that reversibly alters the localization, stability, and biological activity of target molecules. Here, we discuss the available knowledge on the biological roles and underlying mechanisms linked to protein palmitoylation in modulating PRRs and their downstream signaling pathways under physiological and pathological conditions. Moreover, recent advances in the use of palmitoylation as an attractive therapeutic target for disorders caused by the dysregulation of PRRs were summarized.

## Graphical abstract

**Figure:**
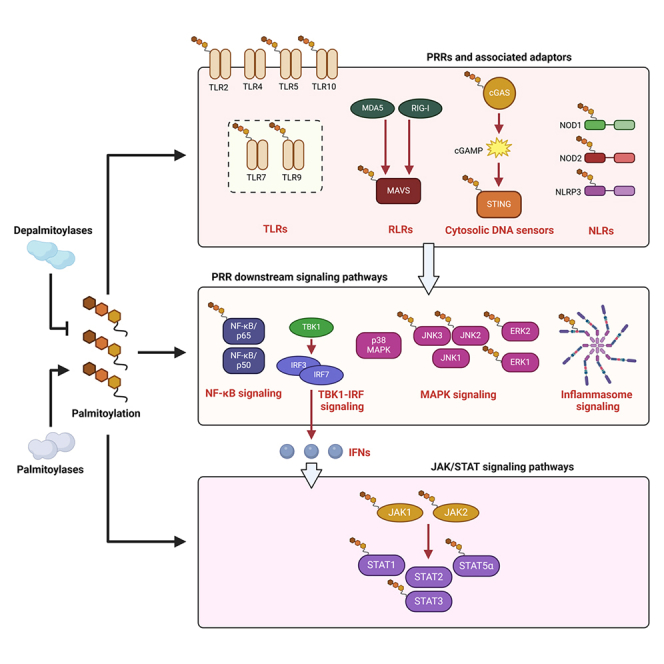

## Introduction

As the first line of host defense, the innate immune system recognizes pathogen-associated molecular patterns (PAMPs) from the outside and damage-associated molecular patterns (DAMPs) in host cells via pattern recognition receptors (PRRs) to maintain physiological homeostasis.^1^^,^^2^ Toll-like receptors (TLRs), nucleotide oligomerization domain (NOD)-like receptors (NLRs), cytosolic DNA sensors, and retinoic acid-inducible gene-I (RIG-I)-like receptors (RLRs) are well-identified subfamilies of PRRs.^3^^,^^4^^,^^5^ After recognizing PAMPs or DAMPs, these PRRs sensitize nuclear factor-κB (NF-κB), mitogen-activated protein kinase (MAPK), TANK-binding kinase 1 (TBK1)-IFN regulatory factor (IRF), and inflammasome signaling pathways,^3^^,^^6^ to elicit the production, as well as secretion, of inflammatory cytokines and interferons (IFNs) to activate Janus kinase/signal transducer and activator of transcription (JAK-STAT) signaling pathways, resulting in anti-infection, antitumor, and other specific immune responses.

Post-translational modifications (PTMs) are covalent or enzyme-dependent modifications that occur at specific amino acid side chains of target proteins. To date, conventional PTMs, including ubiquitination and phosphorylation, and unconventional PTMs, such as acetylation and methylation, have been demonstrated to control PRR-dependent immune responses by controlling but not limited to the activity, stability, subcellular distribution, and aggregation of their innate sensors and downstream signaling molecules.^7^ In addition, increasing attention has been given to palmitoylation, an unconventional PTM that is modified by long-chain fatty acids. With advances in proteomics and bioinformatics, it is estimated that no less than 10% of the human proteome can be modified by palmitoylation, including the molecules that participate in glucose and fat metabolism and inflammation.^8^ In particular, in recent years, palmitoylation has gained broad recognition for its ability to modulate ligand interactions, protein localization, molecular trafficking, protein stability, and signal transduction of core molecules in innate immunity.^8^^,^^9^

More importantly, to date, the subfamilies of PRRs, including TLRs,^10^^,^^11^ RLRs,^12^ cytoplasmic DNA sensors,^13^^,^^14^ NLRs,^15^^,^^16^ and their downstream NF-κB,^17^ MAPK,^18^ inflammasome,^16^ and JAK-STAT signaling pathways,^19^ have been determined to be modulated by palmitoylation to control innate immune responses under physiological conditions, as well as in different diseases, including microbial infection, autoimmune diseases, and tumors. In this review, we focused on the current discoveries regarding the direct or indirect participation of palmitoylation in the regulation of PRRs and their downstream signaling pathways, and discussed the therapeutic potential of targeting palmitoylation to treat disorders caused by aberrant PRR-signaling.

## Palmitoylation

**Figure 1:**
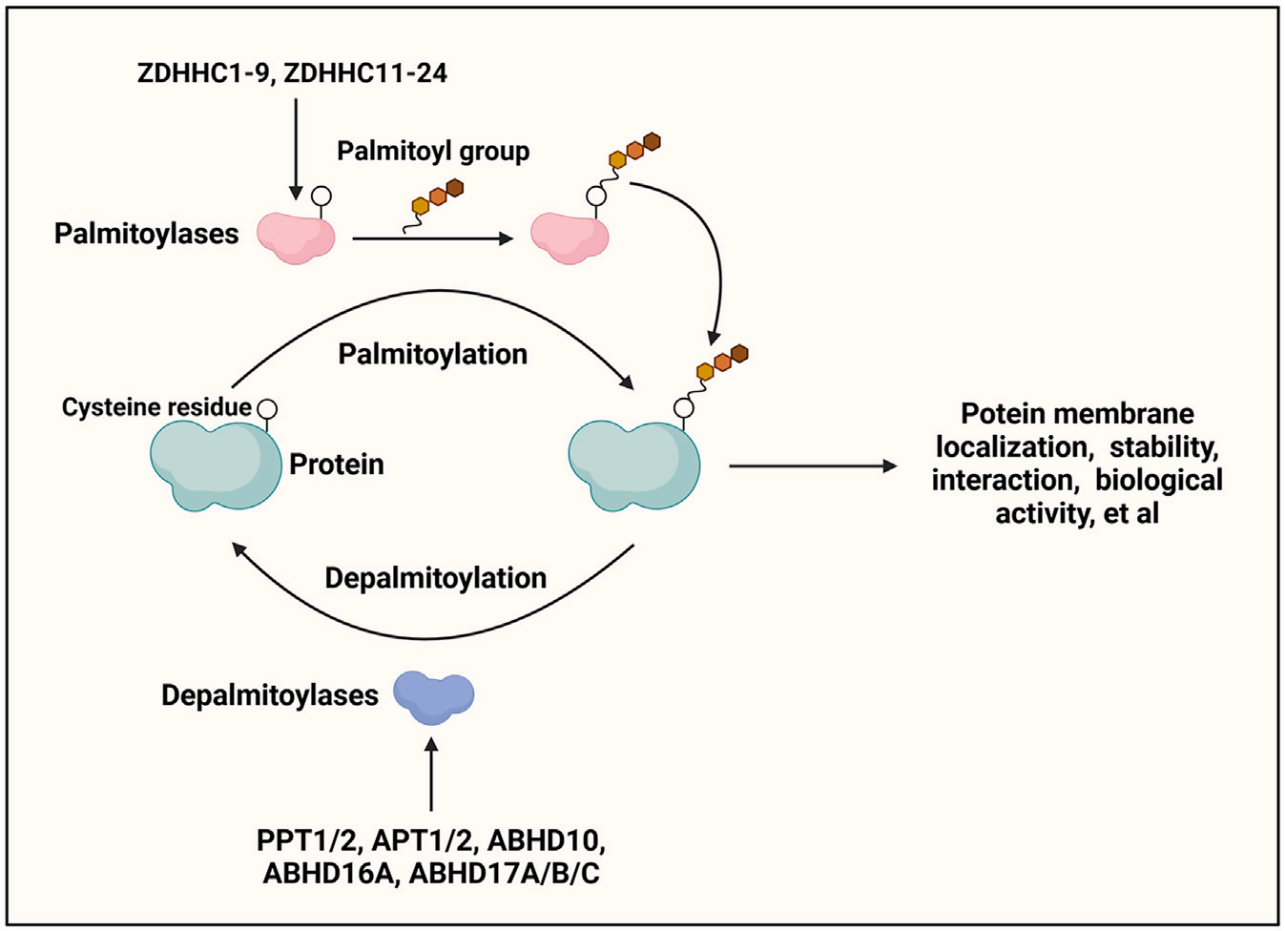
Dynamic protein palmitoylation Palmitoylation is a reversible and dynamic posttranslational protein modification mediated by palmitoylases, such as ZDHHC1-9 and ZDHHC11-24, and depalmitoylases, including PPT1/2, APT1/2, ABHD10, ABHD16A, ABHD17 A/B/C, which attach to or remove palmitoyl groups from protein cysteine residues. ZDHHCs are self-palmitoylated first before transferring the palmitoyl group to substrates. Palmitoylation regulates protein membrane localization, stability, interaction, biological activity et al.

Palmitoylation is called S-palmitoylation or S-acylation. It is a PTM^20^ in which long-chain fatty acids (16 carbon palmitic acid (PA), or named palmitate (C16:0)) are covalently modified to cysteine (Cys) residues via a thioester bond^21^ to increase the functional diversity of target proteins. Emerging evidence reveals that similar to other PTMs, including phosphorylation, methylation, acetylation, and ubiquitination, palmitoylation is a dynamic and reversible process regulated by specific proteases to control protein membrane localization, stability, interaction, biological activity of substrates et al. (Figure 1), including the molecules involved in PRR signaling. In phosphorylation, methylation, and acetylation, chemical groups (phosphoryl group, methyl group, and acetyl group) are added to substrates. In ubiquitination, small proteins (ubiquitin) are covalently attached to target proteins. However, the role of palmitoylation in regulating target proteins, including PRR-signaling-associated proteins, is dependent on the transfer of the palmitoyl group to substrates.^8^^,^^9^^,^^22^ Moreover, dysregulated palmitoylation has been demonstrated to be involved in the pathogenesis of many different diseases, including viral infections, diabetes, cancers, autoimmune diseases, and neurodegenerative disorders.^8^^,^^9^^,^^21^

Although palmitoylation was discovered more than four decades ago, the biological functions and importance of palmitoylation were not well acknowledged until 2002 following the identification of palmitoylases, which opened a new avenue for exploring PTMs.^21^ At present, only one type of mammalian palmitoylase, the ZDHHC family protein, which is characterized by a conserved DHHC (Asp-His-His-Cys) domain with palmitoyl transfer enzyme activity, has been reported.^23^ In mammals, there are 23 ZDHHCs, including ZDHHC1-9 and ZDHHC11-24 (No ZDHHC10). Functionally, they interact with both substrate proteins and the lipid donor palmitoyl-CoA, after which the palmitoyl group is transferred from the palmitoyl-CoA to the DHHC motif in ZDHHC proteins and further to the Cys residues in substrates (Figure 1).^8^^,^^21^

Palmitoylation is a reversible biological process. The palmitoyl group can be removed from the modified proteins by depalmitoylases. At present, a few depalmitoylases, including palmitoyl-protein thioesterase 1/2 (PPT1/2), acyl protein thioesterase 1/2 (APT1/2), α/β hydrolase domain containing 10 (ABHD10), ABHD16A, and ABHD17 A/B/C, have been found to catalyze the removal of palmitoylation.^8^^,^^21^^,^^22^^,^^24^

## Regulation of PRRs mediated by palmitoylation

As mentioned, TLRs, NLRs, cytosolic DNA sensors, and RLRs are the most well-studied PRRs that are capable of inducing host innate responses to endogenous and exogenous stimuli.^4^ In addition to these PRRs, C-type lectin receptors (CLRs) and extracellular soluble pattern recognition molecules, including pentraxin, collectin, and ficolin, are also popular subfamilies of PRRs under study. With the participation of Ca+, CLRs have the capability of recognizing carbohydrates on the surface of pathogens. Unlike cell-related PRRs, extracellular soluble pattern recognition molecules are a vital part of nonspecific humoral immunity and have antibacterial effects on serum.^3^ Because the effects of palmitoylation on CLRs and soluble pattern recognition molecules are still unknown, we focused on recent findings on the modulation of TLRs, NLRs, cytosolic DNA sensors, RLRs, their adaptors, and downstream signaling molecules mediated by palmitoylation.

## Methods of detecting PRR-signaling associated molecule palmitoylation

Until now, to identify palmitoylation, several methodologies, such as radioactive PA metabolic labeling (labeling with [^3^H]-Palmitate or [^125^I]-Iodopalmitate), acyl-biotin exchange (ABE), acyl-resin assisted capture (Acyl-RAC), click chemistry, mass spectrometry (MS), proximity ligation assay (PLA), and PalmPISC have been developed.^25^^,^^26^^,^^27^ In particular, current studies indicate that the approaches, including radioactive labeling with [^3^H]-Palmitate, ABE, Acyl-RAC, click chemistry, and MS, have been used for measuring the palmitoylation of PRR-signaling-associated molecules. When target proteins are labeled with [^3^H]-Palmitate, their palmitoylation can be measured by autoradiography after SDS-PAGE. Based on this method, the stimulator of IFN genes (STING) palmitoylation were measured in different studies.^14^^,^^28^^,^^29^ ABE is dependent on hydroxylamine-induced cleavage of PA followed by biotinylation of Cys residues to detect protein palmitoylation. Acyl-RAC can simplify the ABE process by capturing depalmitoylated proteins based on thiol-reactive resin. Because ABE and acyl-RAC are easier, nonradioactive, and time-saving, these two methods have been widely used to detect the palmitoylation of various molecules, including palmitoylated proteins which locate on the cancer exosome surface and bind to TLR2,^30^ myeloid differentiation primary response gene 88 (Myd88),^31^^,^^32^ cyclic GMP-AMP synthase (cGAS),^13^^,^^33^^,^^34^ STING,^35^ NLR thermal protein domain associated protein 3 (NLRP3),^36^ and NOD2,^37^^,^^38^ in PRR-signaling. Click chemistry is dependent on the use of bioorthogonal reactions with fatty acids to analyze palmitoylated proteins. The approach has been used for detecting palmitoylation of different PRR-signaling molecules TLR9, MyDD8, STING, and NLRP3.^10^^,^^15^^,^^32^^,^^39^ In addition, MS is primarily used for high-throughput identification of palmitoylated proteins and their modification sites. In particular, based on MS, the palmitoylation sites of different molecules, TLR2,^11^ TLR9,^10^ and NLRP3,^40^ in PRR-signaling, have been identified by various groups.

## Palmitoylation with TLRs and their adaptors

TLRs are highly conserved transmembrane glycoproteins that recognize a large variety of PAMPs.^4^ Ten isoforms of TLRs (TLR1-10) have been discovered in humans. TLR1-2, TLR4-6 and TLR10 are located on the cell membrane. TLR3 and TLR7-9 anchor to intracellular endosomes. In response to stimuli, TLRs (excluding TLR3) are capable of recruiting Myd88,^41^ which further sensitizes downstream molecules, including but not limited to tumor necrosis factor receptor-associated factor 6 (TRAF6) and IL-1R-associated kinase 4 (IRAK4), to trigger the sensitization of the NF-κB and MAPK signaling pathways and lead to the production of proinflammatory cytokines. Following stimulation, TLR3 and TLR4 can also recruit TIR domain-containing adaptor-inducing IFN-β (TRIF) to sensitize TBK1 and subsequently activate IRF3/7 (TBK1-IRF signaling), leading to an IFN-mediated immune response.

**Figure 2:**
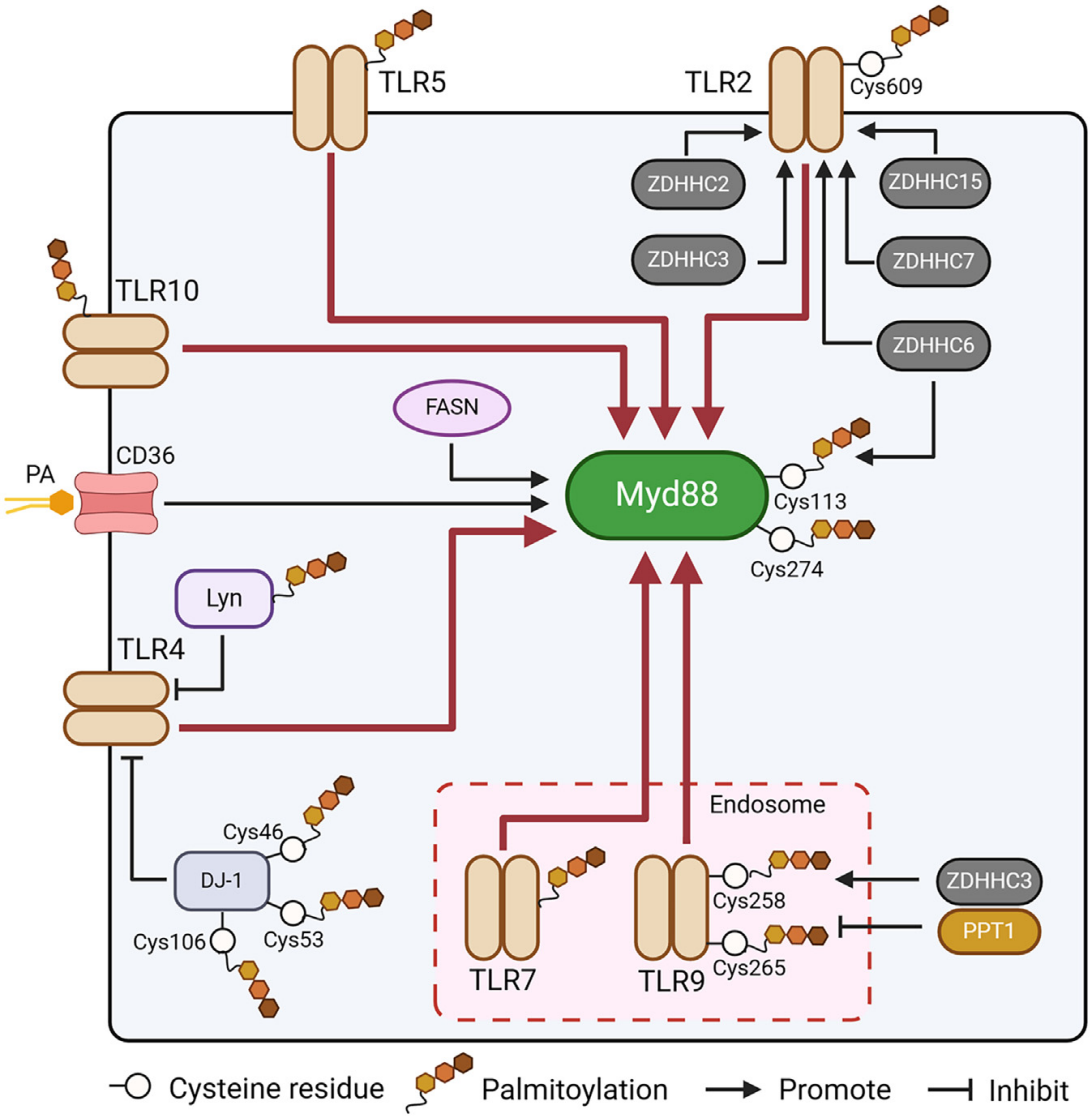
Regulation of TLRs and their adaptors mediated by protein palmitoylation TLR2, TLR5, TLR7, TLR9, and TLR10 can be directly palmitoylated. The palmitoylation site of TLR2 is Cys609. ZDHHC2, ZDHHC3, ZDHHC6, ZDHHC7, and ZDHHC15 are responsible for TLR2 palmitoylation. DJ-1 is palmitoylated at Cys46, Cys53, and Cys106. Palmitoylation of DJ-1 contributes to the abatement of TLR4 activation. Similarly, Lyn palmitoylation hurts TLR4 sensitization. TLR9 can be palmitoylated at Cys258 and Cys265. Palmitoylation of TLR9 is induced by ZDHHC3 but suppressed by PPT1. The palmitoylation sites of Myd88 are Cys113 and Cys274. FASN and CD36 mediated by fatty acids, including PA, induce palmitoylation of the adaptor protein Myd88. ZDHHC6 is the palmitoylase of Myd88. PA: palmitic acid.

To date, palmitoylation modifications have been identified in TLR2, TLR5, TLR7, TLR9, and TLR10.^10^^,^^11^ Among these TLRs, TLR2 can be palmitoylated at Cys609 to facilitate its activation. In particular, palmitoylation is responsible for TLR2-mediated NF-κB signaling-dependent gene upregulation to initiate the inflammatory cytokine response in murine dendritic cells and fibroblasts.^11^ In addition, the inhibition of TLR2 palmitoylation decreases TLR2 surface expression on bone marrow dendritic cells (BMDCs) and reduces the inflammatory response to microbial ligands, including Pam3CSK4, zymosan, and lipomannan. Furthermore, multiple ZDHHCs, including ZDHHC2-3, ZDHHC6-7, and ZDHHC15, have been shown to mediate the palmitoylation of TLR2 (Figure 2).

The activation of TLR2 can also be indirectly regulated by palmitoylation. For example, it has been shown that cancer-secreted exosomes can regulate macrophage activity. In particular, palmitoylated proteins present on the surface of exosomes from human breast cancer cells induce NF-κB signaling activation in macrophages by binding to and activating TLR2 to initiate the generation and secretion of inflammatory cytokines.^30^ Similarly, palmitoylated proteins on acute myeloid leukemia (AML)-derived extracellular vesicles contribute to TLR2 activation to promote myeloid-derived suppressor cell (MDSC) reprogramming.^42^

Current investigation showed that although TLR4 is not a receptor of fatty acids, including PA, the metabolites can activate TLR4 signaling in adipocytes and macrophages to sensitize proinflammatory pathways.^43^^,^^44^^,^^45^ It is speculated that TLR4 may be regulated by PA via palmitoylation to sensitize its signaling. However, whether TLR4 can be directly palmitoylated is still undefined. To date, increasing evidence indicates that the palmitoylation of other proteins can regulate TLR4 activation. For example, kinases of the Src family are critical regulators of TLR4 signaling induced by its ligand lipopolysaccharide (LPS). Lyn, a Src family kinase, can inhibit TLR4 activation. A recent study indicates that the palmitoylation of Lyn is required for the accumulation of this immune molecule in lipid rafts to determine the negative regulation of TLR4 induced by LPS to block the activity of the NF-κB and IRF3 signaling pathways in RAW264 cells.^46^ In addition to Lyn. The DJ-1 protein also modulates LPS-induced TLR4 signaling to control p38 MAPK signaling sensitization via palmitoylation in astrocytes (Figure 2). In detail, DJ-1 is palmitoylated at Cys46, Cys53, and Cys106. Upon stimulation with LPS, the palmitoylation facilitates DJ-1 transport into lipid rafts, in which DJ-1 contributes to TLR4 endocytosis to weaken its activation.^47^

Interestingly, LPS stimulation can induce the palmitoylation of proteins with diverse biological functions, and some palmitoylated proteins are required for the inflammatory response mediated by TLR4 activation. In Kupffer cells, LPS increases the palmitoylation of G protein-coupled receptor kinase 6 (GRK6) to promote its translocation to the cell membrane and induce inflammatory responses, which depend on the activation of the PI3-K signaling pathway. However, inhibition of GRK6 palmitoylation blocks GRK6 membrane translocation and impairs inflammatory reactions mediated by LPS-stimulated TLR4 activation.^48^ In RAW264 cells, LPS also triggers the palmitoylation of several proteins with different biological activities. In particular, LPS-induced palmitoylation of the phosphatidylinositol 4-kinase PI4KIIβ enhance IRF signaling activation mediated by TLR4.^49^

Although TLR7 can be palmitoylated,^10^ the biological role of TLR7 palmitoylation in the modulation of TLR7 signaling has yet not been reported. The palmitoylase ZDHHC2 has been reported to facilitate plasmacytoid dendritic cell (pDC)-induced inflammatory responses in psoriasis, a disease associated with abnormal TLR7 activation.^50^^,^^51^ In particular, in pDCs, ZDHHC2 can enhance IRF7 signaling and IFN-α production by stimulating TLR7 through the ligand imiquimod. Whether ZDHHC2 has a direct impact on TLR7 palmitoylation to control its sensitization is worth investigating in the future.

TLR9 is a DNA sensor that can recognize pathogenic nucleic acids in endosomes. Current research reports that the palmitoylation cycle plays a vital role in systemic lupus erythematosus (SLE). TLR9 can be palmitoylated at Cys258 and Cys265 (Figure 2), and ZDHHC3 is responsible for palmitoylating TLR9 in the Golgi to control the transfer of TLR9 to endosomes. In endosomes, TLR9 also requires UNC93B1 for its stabilization and transport from the endoplasmic reticulum (ER)-Golgi to endolysosomes. In contrast to ZDHHC3, PPT1 causes the depalmitoylation of TLR9. TLR9 depalmitoylation contributes to its release from UNC93B1 in endolysosomes.^10^ In an SLE mouse model, PPT1 was shown to exacerbate disease by removing TLR9 palmitoylation.

In addition to the role of TLR molecules, the palmitoylation of the adaptor protein Myd88 in signaling has also been reported. For example, in mouse models of sepsis, intracellular saturated fatty acids contribute to the activation of Myd88 to facilitate TLR-mediated inflammation.^32^ In particular, the palmitoylation of Myd88 could be controlled not only by fatty acid synthase (FASN), an enzyme responsible for intracellular saturated fatty acid synthesis, but also by CD36, a receptor that controls the incorporation of exogenous fatty acids, including PA. Although Cys113 and Cys274 in Myd88 can be palmitoylated, only palmitoylation at Cys113 in the intermediary domain (INT) domain of the immune molecule is responsible for recruiting IRAK4 and facilitating TLR4 signaling activation. Subsequent investigations revealed that the palmitoylation of Myd88 relies on ZDHHC6 (Figure 2). Blockade of ZDHHC6 with small interfering RNA (siRNA) decreased the palmitoylation of Myd88 and inhibited TLR4 activation upon stimulation with LPS.

Moreover, with LPS stimulation, the palmitoylase ZDHHC11 has been shown to interact with TRAF6, an immune molecule that can complex with Myd88 in the TLR signaling pathway, to enhance its oligomerization and ligase activity, thereby sensitizing IKK complexes to facilitate NF-κB signaling activation in HEK293T cells.^52^ However, whether ZDHCC11 can induce TRAF6 palmitoylation to regulate the immune response mediated by TLRs is unclear.

## Palmitoylation with RLRs and their adaptors

RLRs consist of the double-stranded RNA (dsRNA) sensors RIG-I, melanoma differentiation-associated gene 5 (MDA5), and the laboratory of genetics and physiology-2 (LGP-2). Functionally, these cytoplasmic RNA sensors can recognize nucleic acids from different RNA viruses. Once stimulated, RIG-I and MDA5 bind to and sensitize mitochondrial antiviral signaling (MAVS), an adaptor protein localized on the mitochondrial membrane.^4^^,^^53^ Subsequently, MAVS activates TRAF3/6, thereby recruiting TANK-binding kinase 1 (TBK1) to enhance the activities of the IRF3/7 and NF-κB signaling pathways, leading to IFN and inflammatory cytokine production.

**Figure 3:**
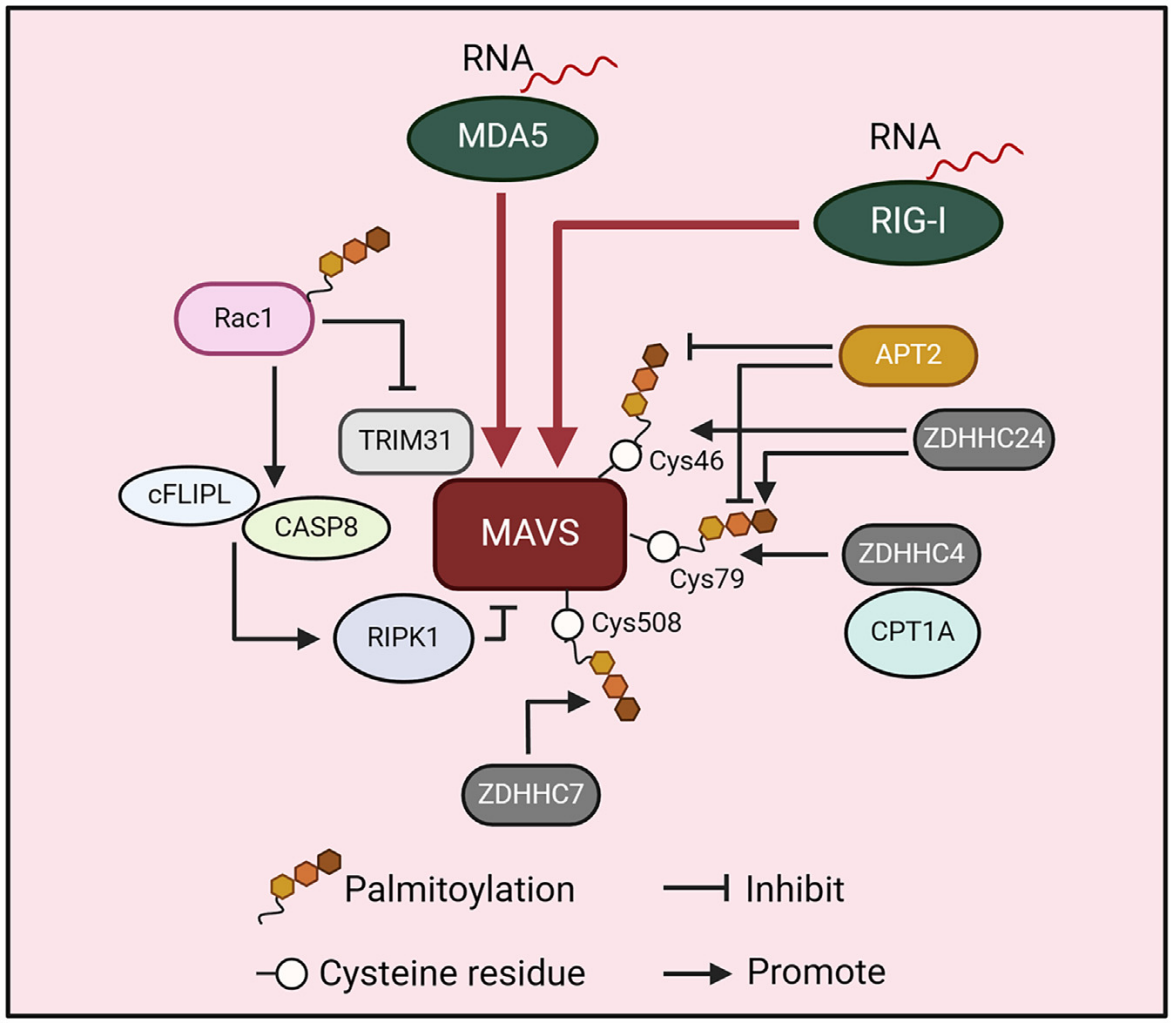
Regulation of the RLR adaptor MAVS mediated by protein palmitoylation In the RLR signaling pathway, CPT1A interacts with ZDHHC4 to promote MAVS palmitoylation at Cys79. ZDHHC24 benefits MAVS palmitoylation, while APT2 depalmitoylates MAVS at Cys46 and Cys79. MAVS could also be palmitoylated by ZDHHC7 at Cys508. In addition, palmitoylation of Rac inhibits MAVS activation by inhibiting the interaction of TRIM31 with MAVS and recruiting cFLIPL and CASP8 to activate RIPK1 cleavage and subsequently inhibit MAVS.

To date, whether RIG-I, MDA5, or LGP-2 can be palmitoylated is still unknown. However, current evidence has demonstrated that the palmitoylation of MAVS is responsible for its stabilization and activation. For example, PA has been demonstrated to induce MAVS palmitoylation at Cys46 and Cys79, thereby facilitating MAVS aggregation and activation to increase innate immune response. During the regulation of MAVS palmitoylation mediated by the fatty acid, ZDHHC24 benefits MAVS palmitoylation. Conversely, APT2 de-palmitoylates MAVS.^54^ During Sendai virus (SeV) infection, as well as vesicular stomatitis virus (VSV) stimulation, to sustain the double-stranded RNA-induced IFN response, mitochondrial carnitine palmitoyltransferase 1A (CPT1A) can recruit ZDHHC4 to the ER via its interaction with ZDHHC4 to promote MAVS palmitoylation at Cys79 in the caspase recruitment domain (CARD) (Figure 3). Subsequently, the palmitoylation of MAVS can promote its stabilization and activation by enhancing K63-associated ubiquitination of MAVS but antagonizes K48-linked ubiquitination to amplify the IFN-dependent immune response and strengthen its antiviral immunity and epigenetic perturbation-induced antitumor effects.^12^ In addition, Lin et al. showed that MAVS could be palmitoylated by ZDHHC7 at Cys508, and the palmitoylation can stabilize MAVS aggregation on the mitochondrial outer membrane and subsequently facilitate antiviral innate immunity.^55^

Yang et al. showed that the activation of MAVS can also be indirectly regulated by protein palmitoylation. During SeV infection, palmitoylation enhances the translocation of Rac1 to mitochondria-associated ER membranes (MAMs) in mice. After localization to MAMs, Rac1 inhibits the binding of MAVS to tripartite motif protein 31 (TRIM31), an E3 ligase, attenuating the ubiquitination, aggregation, and activation of MAVS. In addition, Rac1 contributes to the recruitment of cFLIPL and caspase-8 to MAVS and promotes the cleavage of receptor-interacting protein kinase 1 (RIPK1), thereby terminating MAVS signaling (Figure 3).^56^

## Palmitoylation with cytosolic DNA sensors and their adaptors

Cytosolic DNA sensors, including cGAS, melanoma 2 (AIM2), and DNA-dependent activators of IFN regulatory factors (DAI)/Z-DNA Binding Protein 1 (ZBP1), have been discovered. After cGAS or DAI/ZBP1 is activated, cyclic guanosine monophosphate-adenosine monophosphate (cGAMP) is produced. Subsequently, cGAMP activates STING in the ER.^4^^,^^57^ STING is further transported to the Golgi and binds with TBK1 to trigger IRF3/7 signaling sensitization and stimulate IFN gene transcription. In addition, STING also sensitizes cells to NF-κB signaling to stimulate the expression of inflammatory cytokines.^4^^,^^58^

AIM2 is an AIM2-like receptor that contains four members—AIM2, IFI16, IFIX, and MNDA—in humans.^59^ Unlike cGAS or DAI/ZBP1, AIM2 can recruit apoptosis-associated speck-like protein containing CARD (ASC) and caspase-1 after DNA stimulation to facilitate inflammasome formation, which further contributes to cell pyroptosis, as well as the maturation and release of IL-1β and IL-18.

**Figure 4:**
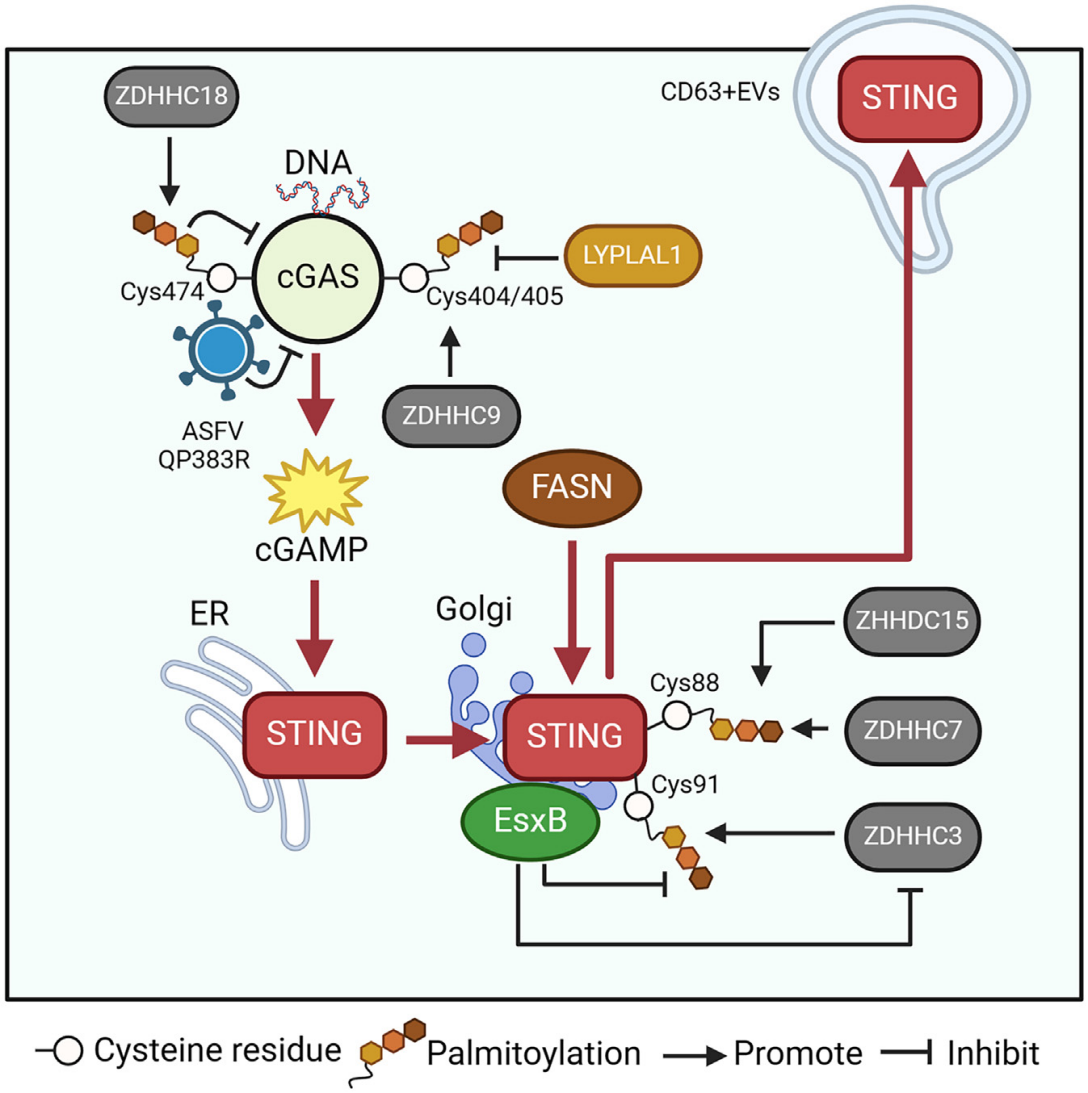
Modulation of cytosolic DNA sensors and their adaptors induced by protein palmitoylation ZDHCC9 induces palmitoylation of the DNA sensor cGAS at Cys404/405 to promote its activation. LYPAL1 inhibits cGAS palmitoylation. ZHDHCC18 enhances cGAS palmitoylation at Cys474 to repress its activation. ASFV QP383R can inhibit cGAS by interacting with cGAS to enhance its palmitoylation. STING can be palmitoylated by FASN. STING is palmitoylated at Cys88 and Cys91. STING palmitoylation facilitates its packaging in CD63^+^ extracellular vesicles (EVs) and exocytosis. ZDHHC3, ZDHHC7, and ZDHHC15 induce palmitoylation of STING. EsxB inhibits palmitoylation of STING at Cys91 by disrupting the interaction between STING and ZDHHC3.

To date, cGAS, but not DAI/ZBP1 or AIM2, has been demonstrated to be palmitoylated upon stimulation with herpes simplex virus 1 (HSV-1) or herring testis DNA (HT-DNA). Furthermore, emerging evidence indicates that palmitoylation of different Cys residues in cGAS has diverse functions. For instance, ZDHHC9 contributes to cGAS palmitoylation at Cys404/405. cGAS palmitoylation at these sites has no effect on its stability, ubiquitination, membrane localization, or transport but facilitates its dimerization, sensitizes STING/TBK1/IRF3 signaling, and causes the transcription of IFN-dependent response genes (Figure 4).^13^ Conversely, lysophospholipase-like 1 (LYPLAL1) is a regulator of cGAS depalmitoylation. Inhibition of LYPLAL1 by short hairpin RNA (shRNA) activates the cGAS-mediated immune response to facilitate antitumor immunotherapy.

Unlike palmitoylation at Cys404/405, palmitoylation of cGAS at Cys474 can restrict its enzymatic activity upon stimulation by HT-DNA in HEK293T cells. cGAS palmitoylation at Cys474 is mainly catalyzed by ZDHHC18 (Figure 4). Furthermore, palmitoylation at Cys474 decreases the interaction of cGAS with its ligand, double-stranded DNA to inhibit cGAS dimerization.^34^ In addition, the African swine fever virus (ASFV) QP383R directly interacts with cGAS and enhances its palmitoylation in HEK239T cells to facilitate infection. It has also been shown that QP383R suppresses the interaction of DNA with cGAS to reduce its dimerization, resulting in the inhibition of cGAS enzymatic functions, a reduction in cGAMP production, and subsequent dampening of IFN-I production.^33^

In addition to cGAS, STING can be palmitoylated by FASN.^60^ Functionally, the palmitoylation of STING is vital for its sensitization.^28^ Suppressing STING palmitoylation abolishes the IFN-dependent immune response. The Cys88 and Cys91 sites are responsible for STING palmitoylation (Figure 4), and the mutation of these palmitoylation sites in STING is not able to induce STING-dependent host defense genes. In addition, ZDHHC3, ZDHHC7, and ZDHHC15 were identified as STING palmitoylases. During *Staphylococcus aureus* infection, extracellular protein B (EsxB) of this bacteria could interact with STING. EsxB-STING binding has an inhibitory role in STING’s palmitoylation at Cys91 by disrupting the interaction between STING with ZDHHC3 to inhibit inflammatory responses.^61^ In response to DNA stimulation, STING translocates from the ER to the Golgi, where it sensitizes TBK1 to the trans-Golgi network (TGN). Current evidence suggests that palmitoylation contributes to the transport of STING from the ER to the TGN. Furthermore, STING clustered in the TGN. The formation of the STING cluster is required for its palmitoylation to enhance TBK1 recruitment and activation.^62^

The location of STING in platelets has also been revealed. STING palmitoylation in platelets facilitates granule secretion from platelets to initiate an inflammatory response. For example, sepsis is a bacterial infection-induced systemic inflammatory response syndrome. The activation of STING in platelets is a vital driver of sepsis-induced pathology in mice.^35^ In particular, dependent on palmitoylation, STING binds to syntaxin binding protein 2 (STXBP2), subsequently facilitating the assembly of soluble N-ethylmaleimide-sensitive fusion protein-attachment protein receptor complexes to enhance granule secretion from platelets and further cause septic thrombosis.

Interestingly, STING is not only located in cells and platelets, as mentioned above but can also be packaged in CD63^+^ extracellular vesicles (EVs) and exocytosed during HSV-1 infection. In particular, STING exocytosis is an HSV-1-mediated process and not a host immune response against infection.^63^ EVs containing STING further participate in antiviral immunity in uninfected cells and suppress HSV-1 infection via an STING-dependent immune response. Moreover, STING palmitoylation at Cys88 and Cys91, together with its trafficking from the ER to the TGN, is required for STING exocytosis (Figure 4).

Additionally, current evidence also demonstrates that in cancer cells, STING palmitoylation can regulate its function without an innate immune response. For instance, the expression of STING is upregulated in renal cell carcinoma (RCC) cells, and STING facilitates cell proliferation in cell models and mouse xenotransplant models. Dependent on STING palmitoylation at Cys88 and Cys91, STING is capable of interacting with mitochondrial calcium transporter voltage-dependent anion channel 2 (VDAC2) to inhibit the increase in reactive oxygen species (ROS) and calcium ions in mitochondria, thereby enhancing mitochondrial function and activating mTORC1/S6K signaling. Moreover, suppressing the STING palmitoylases ZDHHC3 and ZDHHC7 reduces the binding of STING to VDAC2 to suppress the growth of RCC cells.^64^

## Palmitoylation with NLRs

NLRs are multidomain proteins composed of a leucine-rich repeat (LRR), a nucleotide-binding domain (NBD), and an effector domain.^65^^,^^66^ The NLR family comprises five subfamilies: NLRA, NLRB, NLRC, NLRP, and NLRX. Among these subfamilies, the NLRP and NLRC subfamilies are the most studied. NLRP has 14 members (NLRP 1–14), and NLRP3 is the most well-studied.^66^ After the recognition of exogenous pathogens and endogenous danger signals, NLRP can form inflammasomes by recruiting ASC and caspase-1. Inflammasome signaling subsequently initiates an inflammatory response.^67^ The NLRC is composed of 5 members (NLRC 1–5). The well-identified NLRCs are NLRC1 and NLRC2, which are also called NOD1 and NOD2.^66^ After stimulation, NOD1 and NOD2 can activate NF-kB signaling.

**Figure 5:**
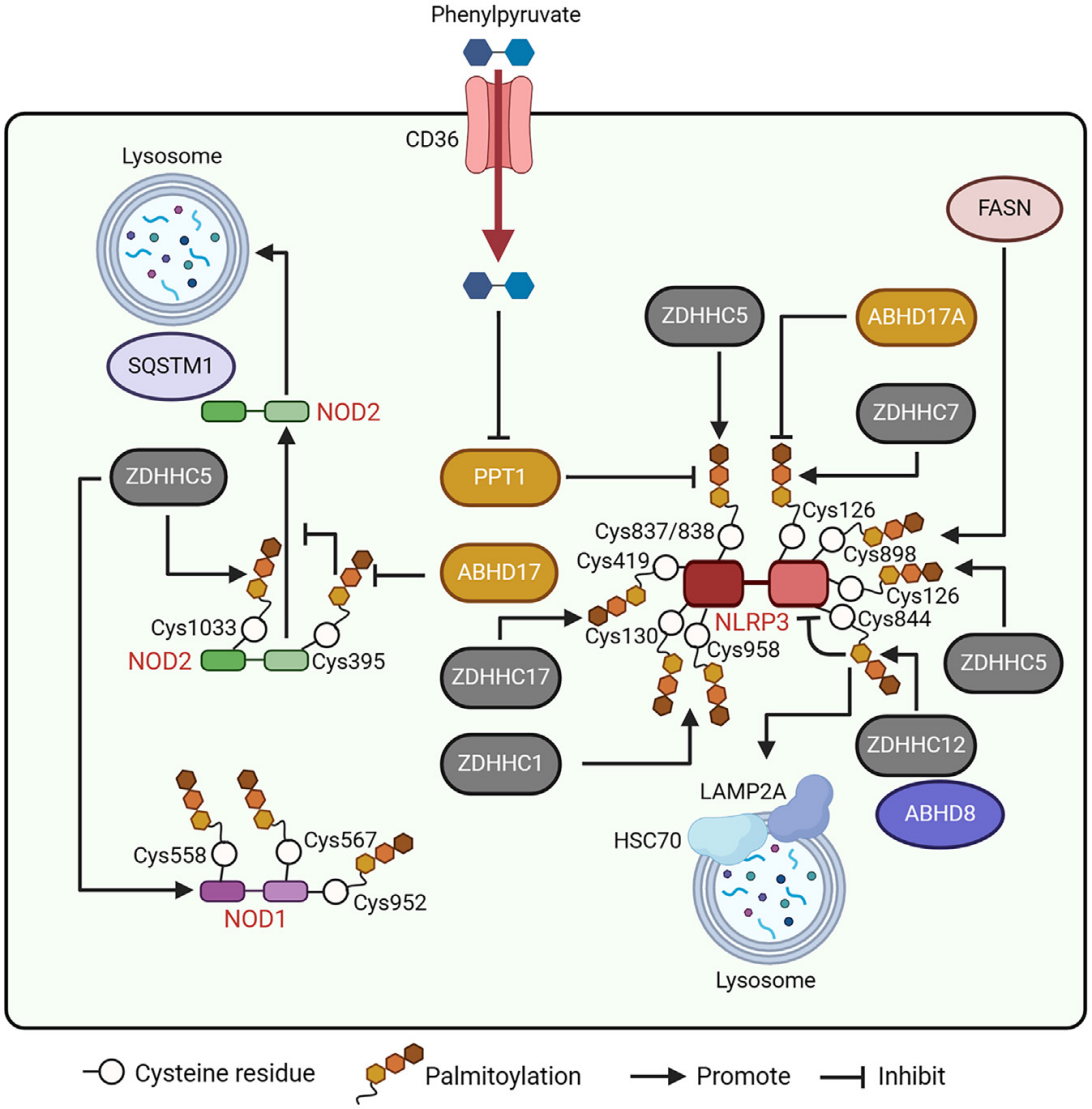
Modulation of RLRs induced by protein palmitoylation NLRP3 palmitoylation is induced by ZDHHC7 but suppressed by ABHD17A at Cys126. ZDHHC5 induces NLRP3 palmitoylation at Cys837/838. Palmitoylation of NLRP3 at Cys130 and Cys958 is regulated by ZDHHC1. Based on ZDHHC17, NLRP3 is palmitoylated at Cys419. Conversely, PPT1 suppresses NLRP3 palmitoylation. CD36 mediates the entry of phenylpyruvate into macrophages to inhibit PPT1 and promote NLRP3 palmitoylation. FASN enhances the palmitoylation of NLRP3 at Cys898. ZDHCC12 contributes to NLRP3 palmitoylation at Cys844 to inhibit its expression by improving NLRP3 degradation via the HSC70 and LAMP2A-associated autophagy-lysosomal pathways. In addition, ABHD8 can recruit ZDHHC12 to NLRP3 to enhance NLRP3 degradation. NOD1 is palmitoylated at Cys558, Cys567, and Cys952. NOD2 is palmitoylated at Cys395 and Cys1033. ZDHHC5 contributes to palmitoylation of both NOD1 and NOD2. Palmitoylation of NOD2 blocks its degradation via the SQSTM1-associated autophagy-lysosomal pathway. However, ABHD17 inhibits NOD2 protein stabilization by reducing NOD2 palmitoylation.

The palmitoylation of NLRP3, a well-known inflammasome-linked NLR, has been demonstrated. Furthermore, multiple palmitoylation sites in NLRP3 have been reported by different groups. Interestingly, the roles of NLRP3 palmitoylation at different sites are diverse. For example, ZDHHC5 induces the palmitoylation of NLRP3 at Cys837/838 in the leucine-rich repeat (LRR) domain to facilitate its activation. Conversely, ABHD17A depalmitoylates NLRP3 (Figure 5). In particular, in human cells and mice, silencing ZDHHC5 can inhibit NLRP3 oligomerization, block the binding of NLRP3 to NIMA-related kinase 7 (NEK7),^40^ and suppress the formation of oligomeric ASCs to abolish the release of IL-1β and IL-18. Wang et al. showed that based on ZDHHC17, NLRP3 can be palmitoylated at Cys419 to enhance NLRP3 activation by promoting the interaction between NEK7 with NLRP3.^68^ Xu et al. found that ZDHHC5 activates NLRP3 by promoting its palmitoylation at Cys126.^69^ In addition, palmitoylation of NLRP3 at Cys130 and Cys958 regulated by ZDHHC1 is critical for its membrane trafficking to microtubule-organizing center (MTOC), where large tumor suppressor kinases 1 and 2 (LATS1/2)-mediated phosphorylation of NLRP3 facilitates its interaction with NEK7 and subsequently inflammasome activation.^70^

ZDHHC7 also palmitoylates NLRP3 at Cys126 in macrophages. ZDHHC7-mediated NLRP3 palmitoylation is essential for immune molecule localization to the TGN. Moreover, palmitoylation at Cys126 in NLRP3 is vital for the recruitment of ASC and subsequent inflammasome assembly.^15^ In addition, in a CD36-dependent manner, phenylpyruvate, a metabolite of phenylalanine, can be taken up by macrophages. In macrophages, phenylpyruvate interacts with PPT1 to inhibit its enzyme activity and increase NLRP3 palmitoylation levels (Figure 5). In particular, increased NLRP3 palmitoylation enhances its protein stability to increase NLRP3 inflammasome signaling activation to inhibit diabetic wound healing in mice.^71^

Current evidence also demonstrates that the fatty acid biosynthesis pathway has a vital role in NLRP3 inflammasome activation. In particular, blockade of FASN, a vital enzyme in the pathway, can abrogate NLRP3 activation in mouse and human macrophages.^72^ Moreover, Leishman et al. reported that FASN-mediated sensitization of NLRP3 relies on the palmitoylation at Cys898 in the molecule (Figure 5). Especially, palmitoylation at Cys898 enables NLRP3 translocate to dispersed trans-Golgi network (dTGN) vesicles to promote inflammasome assembly and activation.

ZDHHC12 interacts with NLRP3 to promote its palmitoylation in both THP-1 cells and HEK293T cells. However, ZDHHC12-mediated palmitoylation at Cys844 in NLRP3 does not affect its activation but inhibits its expression through heat shock cognate protein of 70 kDa (HSC70) combined with lysosome-associated membrane protein 2A (LAMP2A)-mediated autophagic degradation. Moreover, the ZDHHC12-mediated palmitoylation of NLRP3 at Cys844 is critical for blocking inflammasome activation (Figure 5).^36^ In contrast, defective NLRP3 palmitoylation at this site is closely linked to excessive inflammation in mice. Interestingly, (α/β-hydrolase domain-containing) enzyme (ABHD) 8 can act as a scaffold protein with the capacity to recruit ZDHHC12 to NLRP3 for its palmitoylation and facilitate chaperone-mediated autophagy-mediated NLRP3 degradation. Conversely, ABHD8 deficiency enhances the stabilization of NLRP3 and increases NLRP3 inflammasome activation.^73^

In addition to NLRP3, NOD1/2 has been shown to undergo palmitoylation, which contributes to NOD1/2 activation. Several Cys residues, including Cys567, Cys558, and Cys952 in NOD1 and Cys395 and Cys1033 in NOD2, can be palmitoylated. Moreover, NOD1/2 palmitoylation is vital for membrane recruitment to facilitate the sensitization of NF-κB signaling.^74^ ZDHHC5 has been identified as the palmitoylase of NOD1/2 and ZDHHC5-induced palmitoylation of NOD1/2 benefits immune responses to peptidoglycans. Besides these, palmitoylation mediated by ZDHHC5 contributes to the membrane recruitment and activation of NOD2 by suppressing its autophagic degradation, which is dependent on the selective autophagy receptor Sequestosome-1 (SQSTM1) to enhance NOD2 stabilization (Figure 5).^38^ Furthermore, ZDHHC5 competes with SQSTM1 to interact with NOD2. Interestingly, in patients with the NOD2R444C variant, the ZDHHC5-mediated palmitoylation of NOD2 is enhanced owing to the amplified interaction between these two proteins. Conversely, ABHD17 attenuates the palmitoylation of NOD2^37^ to inhibit its membrane localization and signal transduction in epithelial cells.

## The regulation of the downstream signaling pathways of PRRs by palmitoylation

To initiate immune responses, the recognition of PAMPs or DAMPs by PRRs can induce the sensitization of a variety of conserved intracellular signaling pathways. For example, following ligand engagement, TLRs not only induce the NF-κB and MAPK signaling pathways to increase the production of proinflammatory cytokines but also initiate the TBK1-IRF signaling-dependent expression of IFNs.^75^ Upon stimulation, RLRs sensitize the TBK1-IRF and NF-κB signaling pathways, leading to the gene transcription of IFNs and proinflammatory cytokines. Similar to RLRs, STING activation mediated by the cytosolic DNA sensors DAI/ZBP1 or cGAS can also induce the sensitization of the TBK1-IRF and NF-κB signaling pathways.^58^ In addition, the innate immune responses controlled by NLRs are dependent mainly on the NF-κB or inflammasome signaling pathways.^66^ Except for TBK1-IRF signaling, which is mainly mediated by TBK1 and its downstream transcription factors IRF3/7^3^^,^^4^, palmitoylation is necessary for the activation of PRR-signaling pathways. Here, we summarize up-to-date information on the modulation of the NF-κB, MAPK, and inflammasome signaling pathways mediated by palmitoylation.

## NF-ΚB signaling

NF-κB is a significant downstream transcription factor of PRRs. In the cytosol, without activation signals, the inhibitory molecules IκB-α and IκB-β interact with NF-κB p50/p65 dimers and constitute an inactivation protein complex.^76^ After receiving activation signals from the IKK complex formed by IKKα and IKKβ, the inhibitory molecules IκB-α/IκB-β are degraded in a ubiquitin-dependent proteasome manner. Subsequently, p65/p50 and c-Rel/p50 are free from the inactivation protein complex and transported into the cell nucleus to stimulate proinflammatory cytokine transcription.^4^^,^^77^

**Figure 6:**
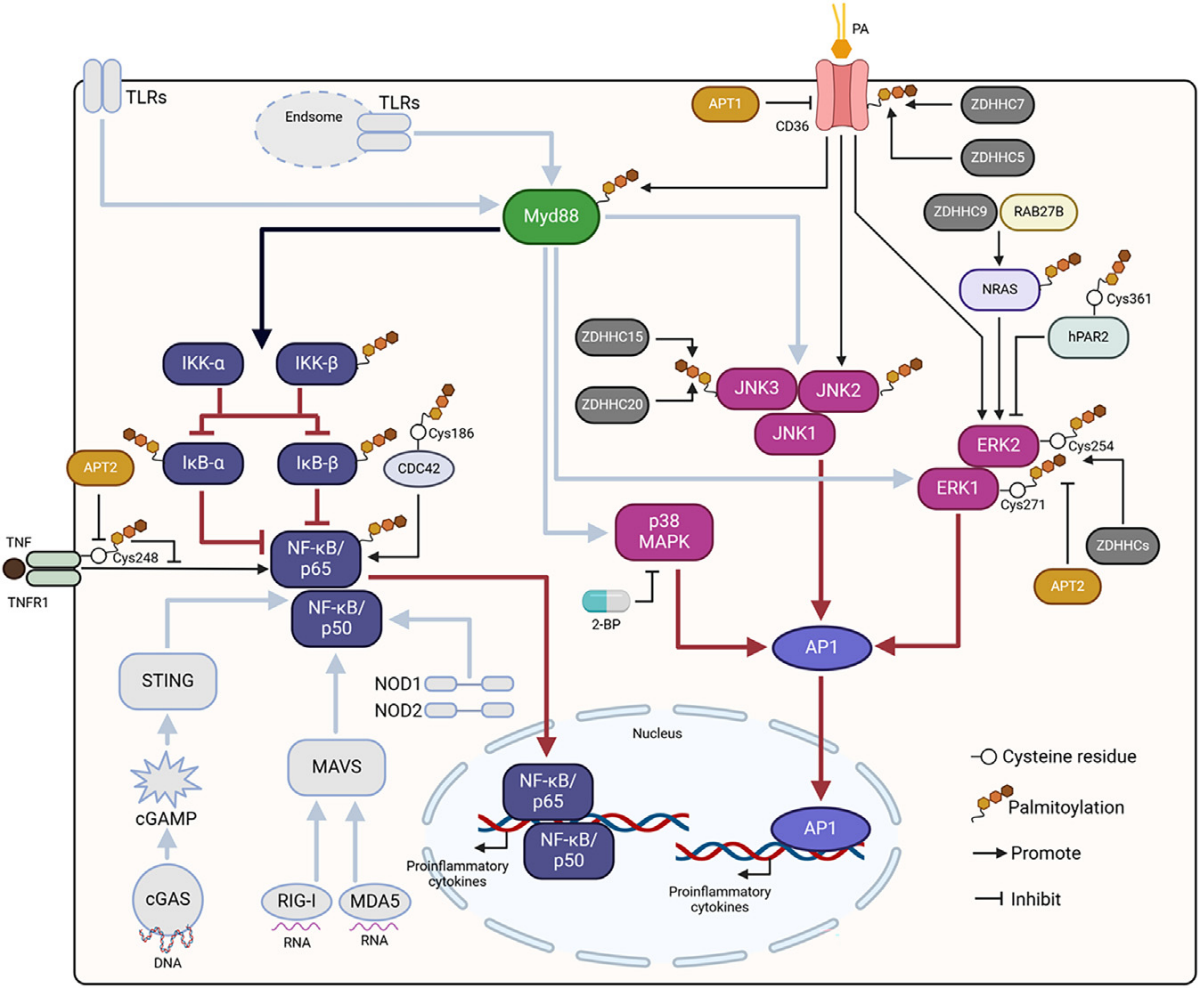
Control of the downstream NF-κB and MAPK signaling pathways of PRRs mediated by protein palmitoylation TLRs (without TLR3) are located on the membrane and endosomes can activate the adaptor protein Myd88 to activate the NF-κB and MAPK signaling pathways. Similar to TLRs, cytosolic DNA sensors, NLRs, and the components of RLRs, including NOD1 and NOD2, also could sensitize NF-κB signaling. During NF-κB signaling, p65, IκB-α, IκB-β, and IKK-β can be palmitoylated. The palmitoylation of CDC42 at Cys186 facilitates NF-κB signaling activation. TNFR1 palmitoylation at Cys248 inhibits NF-κB signaling. However, APT2 can suppress TNFR1 palmitoylation to promote signaling activation in NF-κB signaling. In addition, Myd88 palmitoylation mediated by the activation of CD36 with PA can sensitize NF-κB signaling. In MAPK signaling, ERK1, ERK2, JNK2, and JNK3 can be directly palmitoylated. Although it is unknown whether p38 MAPK proteins can be directly palmitoylated, the palmitoylation inhibitor 2-BP can inhibit p38 MAPK signaling activation. In addition, the palmitoylation site of ERK1 is Cys271, and ERK2 palmitoylation site is Cys254. Moreover, multiple ZDHHCs are beneficial for palmitoylation of ERK1/2. In contrast, APT2 depalmitoylates ERK1/2. The interaction of RAB27B with ZDHHC9 contributes to MRAS palmitoylation to activate ERK signaling. However, hPAR2 palmitoylation at Cys361 represses ERK signaling. CD36 palmitoylation in hepatocytes facilitates JNK signaling activation. In adipocytes, CD36 palmitoylation is promoted by ZDHHC5 and ZDHHC7 but inhibited by APT1, which facilitates the activation of ERK signaling. PA: palmitic acid.

Palmitoylation of p65, IκB-α, and IκB-β, as well as the upstream molecule IKK-β, was detected in the mouse testis via palm-proteomics (Figure 6).^17^ In human retinal pigment epithelial cells, IKKβ palmitoylation has also been identified by proteomic analysis.^78^ However, the role of palmitoylation on these core molecules in the NF-κB signaling pathway is still unclear. In addition, the molecular mechanisms underlying the palmitoylation of these molecules mediated by palmitoylases and depalmitoylases have not yet been defined.

Nevertheless, recent studies have shown that different palmitoylated proteins can regulate NF-κB signaling activation. For example, in human monocytic U937 cells, the palmitoylation of tumor necrosis factor (TNF) receptor 1 (TNFR1) at Cys248 (Figure 6) has been identified.^79^ Functionally, palmitoylation can inhibit TNFR1 localization to the plasma membrane to repress TNF-mediated NF-κB activation. However, APT2 blocks TNFR1 palmitoylation to promote the activation of NF-κB signaling. In addition to TNFR1, the palmitoylation of CDC42 at Cys186 in fibroblasts facilitates the retention of the protein in the Golgi apparatus to enhance NF-κB signaling activation.^80^ However, the detailed mechanisms responsible for the sensitization of NF-κB signaling controlled by Cdc42 palmitoylation have not been well investigated. Besides these, Myd88 palmitoylation mediated by CD36 with palmitate in AML sensitizes NF-kB signaling to drive immunosuppressive gene expression, thereby blocking anti-tumor T cell responses and resistances to hypomethylating agent therapy.^81^

## MAPK signaling

The MAPK family comprises a large number of kinases. Three main classical MAPK subfamilies, ERKs (ERK1 and ERK2), c-Jun N-terminal kinases (JNKs), including JNK1-3, and p38 MAPKs (p38α, p38β, p38γ, and p38δ), have been well studied.^82^ Upon stimulation, these MAPK signaling pathways initiate the activation of the transcription factor AP1 and induce the production of proinflammatory cytokines. Here, we mainly discuss the current knowledge about the effect of palmitoylation on the modulation of ERKs, JNKs, and p38 MAPK in MAPK signaling pathways.

The palmitoylation of ERK1/2 has been shown to facilitate their activation. Conversely, perturbation of ERK1/2 palmitoylation disrupts their transcriptional program. A detailed investigation indicated that the palmitoylation site of ERK1 is Cys271, and EKR2 palmitoylation site is Cys254 (Figure 6). Interestingly, multiple ZDHHCs, including 2, 3, 7, 9, 11, 23, 12, 14, 15, 17, 20, 21, and 25, can increase ERK1/2 palmitoylation. However, only APT2 has been shown to depalmitoylate ERK1/2.^83^ In addition, ERK1/2 activation is indirectly regulated by palmitoylation. For example, human proteinase-activated receptor-2 (hPAR2) is a proteolytically activated G protein-coupled receptor. The protein can be palmitoylated at Cys361, and the hPAR2 C361A mutation promotes ERK activation in Pro5 cells, highlighting the critical role of hPAR2 palmitoylation in the modulation of ERK1/2 signaling.^84^ In leukemia cells, RAB27B, a RAB family small GTPase, promotes the palmitoylation of NRAS, and the effect of RAB27B on NRAS palmitoylation relies on ZDHHC9, which interacts with RAB27B. By regulating NRAS palmitoylation, RAB27B facilitates ERK signaling activation to promote leukemia development.^85^ In addition, conjugated linoleic acid (CLA) decreased intestinal fatty acid uptake and chylomicron formation in high-fat diet (HFD)-fed mice by inhibiting CD36 palmitoylation mediated by ZDHHC7. Functionally, the suppression of CD36 palmitoylation decreases ERK signaling activation.^86^

The activation of JNK signaling is also dependent on palmitoylation. JNK2 and JNK3, but not JNK1, can be endogenously palmitoylated in dorsal root ganglion (DRG) neurons (Figure 6).^18^ Furthermore, ZDHHC15 and ZDHHC20 were shown to promote JNK3 palmitoylation. Functionally, palmitoylation regulates the distribution of JNK3 on the actin cytoskeleton.^87^ The indirect modulation of JNK signaling mediated by palmitoylation has also been reported. When fatty acids (FAs) are transported into adipocytes, FAs can bind to CD36 to trigger its internalization to facilitate the transfer of FAs into adipocytes. In detail, the binding of FAs to CD36 sensitizes Lyn, which further phosphorylates ZDHHC5 at Tyr91 to suppress palmitoylase activity and block the palmitoylation of CD36, leading to subsequent depalmitoylation of CD36 by APT1 to recruit the tyrosine kinase Syk and activate JNK.^88^ However, in HepG2 cells, inhibition of CD36 palmitoylation was found to attenuate JNK signaling activation and subsequently reduce the inflammatory response,^89^ suggesting that in different types of cells, the role of CD36 palmitoylation in regulating JNK signaling is diverse.

In addition to ERK and JNK signaling, p38 MAPK sensitization can be suppressed by the palmitoylase inhibitor 2-bromopalmitic acid (2-BP),^90^^,^^91^ indicating that palmitoylation is also necessary for activating signaling. However, whether p38 MAPK proteins can be directly palmitoylated is still unclear, and more work in the future is warranted.

## Inflammasome signaling

After activation by ligands, NLRP can form an inflammasome.^65^ NRLP3-associated inflammasome signaling is the most well-investigated.^65^^,^^67^^,^^92^ Upon stimulation, NLRP3 recruits ASC to induce the formation of an ASC prion-like oligomer, which further interacts with pro-caspase-1 to form an inflammasome and activate caspase-1. Then, caspase-1 cleaves gasdermin D (GSDMD) into the N-terminal fragment GSDMD (GSDMD-NT), and the proinflammatory cytokines pro-IL1β and pro-IL18 are cleaved into IL1β and IL18. Subsequently, GSDMD-NT is transported to the membrane, where it further oligomerizes and forms GSDMD pores, leading to cell pyroptosis and the release of IL1β and IL18 to initiate immune responses.^66^^,^^67^

**Figure 7:**
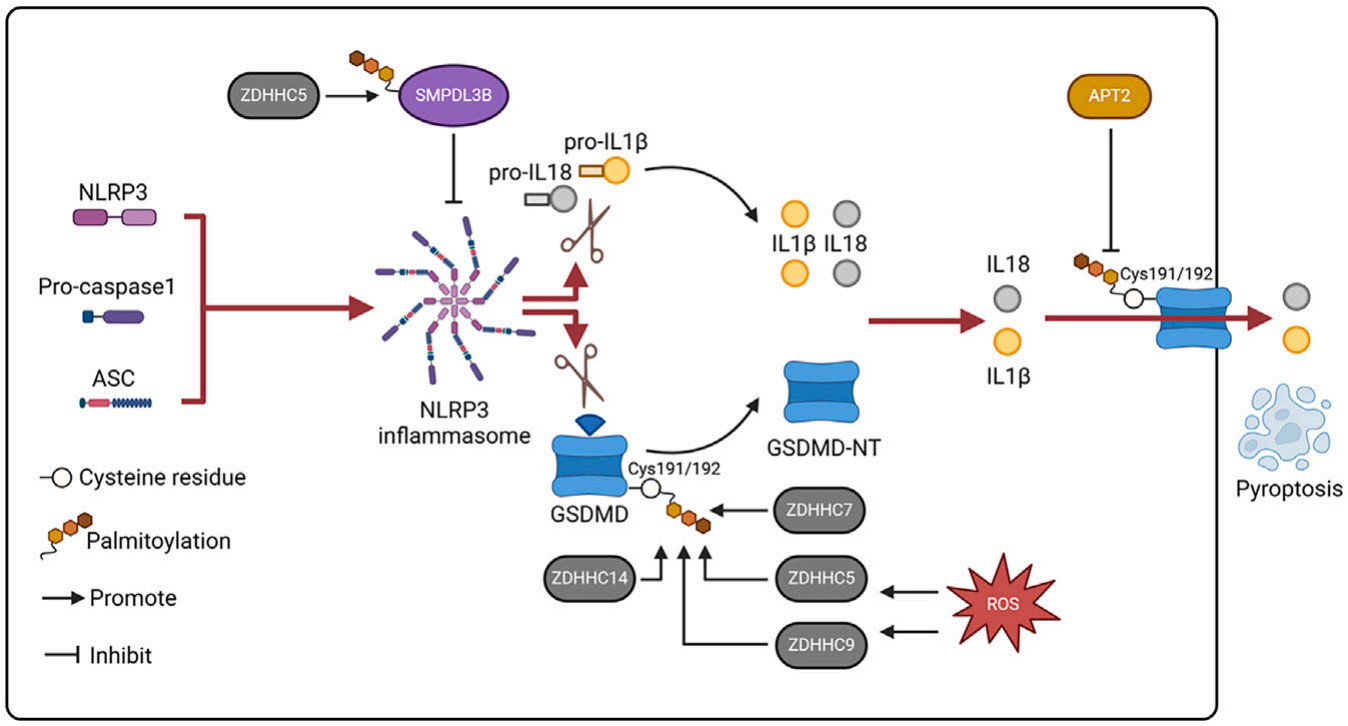
Regulation of the NLRP3 inflammasome signaling pathways of PRRs mediated by protein palmitoylation After stimulation, NLRP3 recruits ASC and forms an ASC prion-like oligomer to interact with pro-caspase-1 to be composed of an inflammasome, leading to caspase-1 activation. Caspase-1 cleaves gasdermin D (GSDMD) and the proinflammatory cytokines pro-IL1β and pro-IL18, causing the production of the N-terminal fragment GSDMD (GSDMD-NT), IL1β and IL18. Then, GSDMD-NT oligomerizes and forms GSDMD pores in the membrane, leading to the release of IL1β and IL18 and the induction of cell pyroptosis. GSDMD can be palmitoylated at Cys191/Cys192 (human/mouse) to facilitate its cleavage, and its transport to the membrane. ZDHHC5, ZDHHC7, ZDHHC9, and ZDHHC14 have been shown to act as palmitoylases of GSDMD-NT. APT2 inhibits palmitoylation of GSDMD-NT on the membrane, promoting GSDMD oligomerization and the formation of GSDMD pores. In addition, SMPDL38 palmitoylation controlled by ZDHHC5 inhibits NLRP3 inflammasome activation.

To date, no signal for palmitoylation has been detected for ASC or caspase-1.^36^ However, during NRLP3 inflammasome signaling activation, palmitoylation of GSDMD has been well demonstrated.^16^^,^^93^ In particular, palmitoylation of GSDMD at Cys191 mediated by ZDHHC7 was identified, and palmitoylation contributed to GSDMD-mediated pyroptosis and cytokine release.^94^ GSDMD palmitoylation at Cys192 in mice facilitates its cleavage mediated by caspases to form GSDMD-NT, which is further transported to the cytomembrane. In addition, Cys192 palmitoylation also mediates GSDMD membrane binding.^95^ Next, APT2 degrades GSDMD-NT on the membrane, promoting its oligomerization (Figure 7).^96^ Another study showed that the palmitoylation of GSDMD could be induced by reactive oxygen species (ROS), and ZDHHC5 and ZDHHC9 regulate its palmitoylation at Cys191/192 (human/mouse) to control membrane translocation and pore formation.^97^^,^^98^ Similarly, Du et al. reported that ZDHHC5 and ZDHHC9 are major palmitoylases that control GSDMD palmitoylation. However, an investigation by Du et al. suggested that GSDMD palmitoylation induced by ROS at Cys191 did not affect its cleavage but promoted pore formation.^99^ In addition to ZDHHC5, ZDHHC7, and ZDHHC9, ZDHHC14 has also been identified as a vital enzyme for GSDMD palmitoylation.^100^

Indirectly, protein palmitoylation also modulates NLRP3 inflammasome signaling. For example, sphingomyelin phosphodiesterase acid-like 3B (SMPDL3B), a vital lipid raft enzyme, is reported to have anti-inflammatory potential. The function and mechanism of SMPDL3B in diabetic retinopathy (DR)-related retinal endothelial injury in mice has been assessed by Zhou et al. This investigation indicated that during DR, SMPDL3B is overexpressed. Moreover, SMPDL3B palmitoylation catalyzed by ZDHHC5 contributes to an increase in SMPDL3B protein expression,^101^ although the palmitoylation sites in SMPDL3B mediated by ZDHHC5 have not been identified. Importantly, SMPDL3B plays a protective role in retinal vascular endothelial dysfunction by suppressing the sensitization of NLRP3 signaling (Figure 7).

## JAK-STAT signaling

In the JAK-STAT signaling pathway, four molecules, including JAK1-3 and TYK2, were discovered in the JAK family. The STAT family comprises seven members (STAT1-4, STAT5α-β, and STAT6).^4^ JAK-STAT signaling can be sensitized by IFN receptors, the activity of which relies on the interaction of IFN, a downstream cytokine of PRR-signaling, to generate IFN-stimulated genes (ISGs).^102^

**Figure 8:**
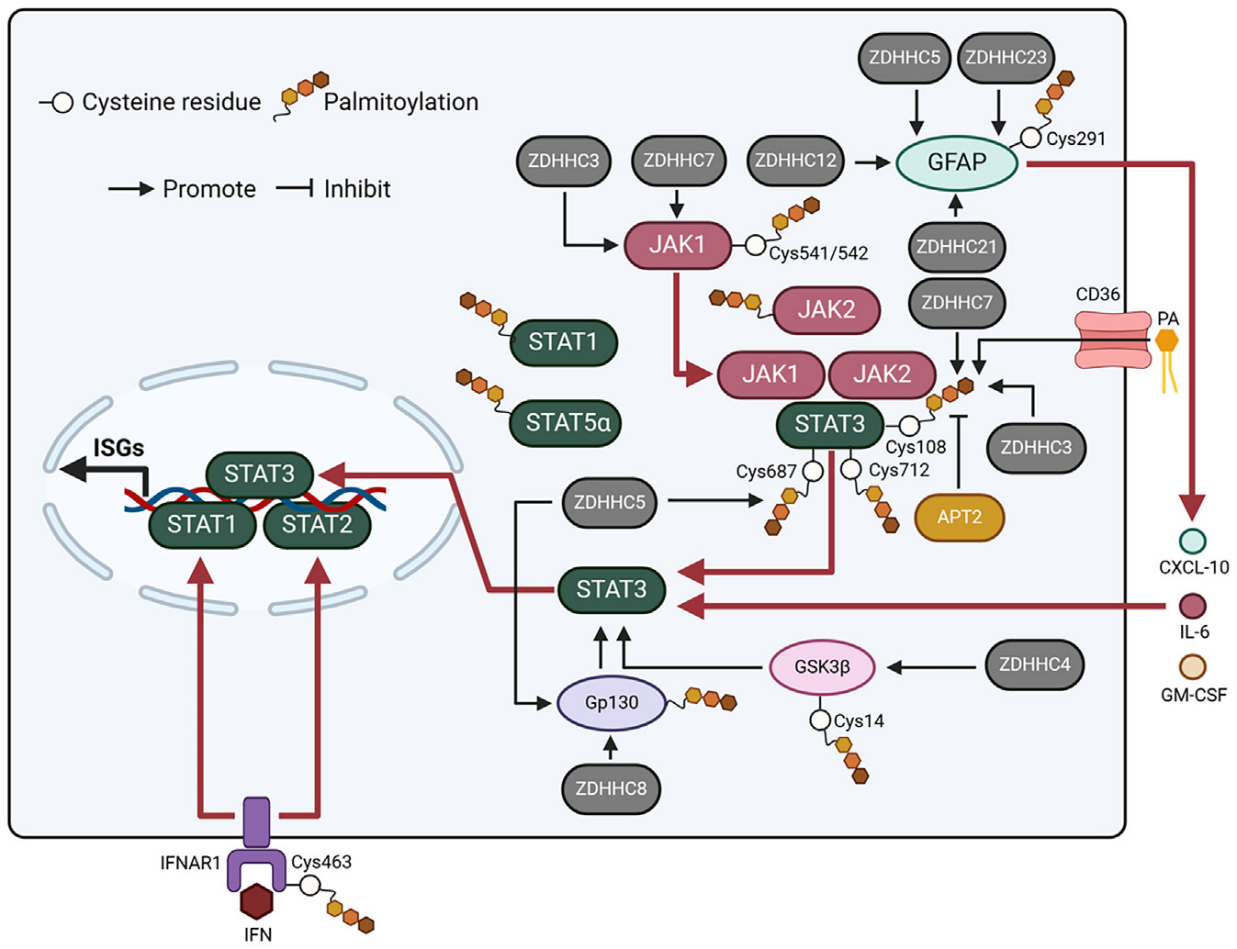
The modulation of JAK-STAT signaling by palmitoylation In the JAK-STAT signaling pathway, JAK1, JAK2, STAT1, STAT3, and STAT5α can be palmitoylated. ZDHCC3 and ZDHHC7 can palmitoylate JAK1 at Cys541/542 to facilitate its activation. IFNAR1 palmitoylation at Cys463 benefits STAT1 and STAT2 activation. PA mediated by CD36 control STAT3 palmitoylation. ZDHHC3 and ZDHHC7 promote STAT3 palmitoylation at Cys108. ZDHHC5 increases STAT3 palmitoylation at Cys687 and Cys712. Gp130 palmitoylation mediated by ZDHHC5 and ZDHHC8, and GSK3β palmitoylation at Cys14 mediated by ZDHHC4 contribute to STAT3 activation. In addition, GFAP can be palmitoylated by ZDHHC5, ZDHHC12, ZDHHC21, and ZDHHC23. GFAP palmitoylation at Cys291 mediated by ZDHHC23 facilitates CXCL-10, IL-6, and GM-CSF secretion to activate STAT3. APT2 depalmitoylates STAT3. PA: palmitic acid.

To date, JAK1, JAK2, STAT1, STAT3, and STAT5α in the JAK-STAT pathway have been demonstrated to be palmitoylated in adipose cells.^19^ The palmitoylation site of JAK1 is Cys541/542. Furthermore, palmitoylation of JAK1 facilitates its binding to the plasma membrane, promoting its activation in adipose cells. Hernandez et al. assessed the role of palmitoylation in JAK1 in neurons. They found that palmitoylation is required for JAK1-dependent signaling.^103^ On the one hand, palmitoylation contributes to JAK1 phosphorylation. On the other hand, palmitoylation of JAK1 is essential for its kinase activity to phosphorylate STAT3. In addition, ZDHHC3 and ZDHHC7 are discovered to act as regulators of JAK1 palmitoylation in neuron cells (Figure 8).

Although it is unknown whether the palmitoylation of STAT1 and STAT2 contributes to their activation, the palmitoylation of other proteins could regulate STAT1 and STAT2 activation. For instance, Claudinon et al. showed that IFN-alpha receptor 1 (IFNAR1) and IFNAR2 could be palmitoylated.^104^ The site of IFNAR1 palmitoylation is Cys463. Although IFNAR1 palmitoylation is not necessary for its endocytosis, protein stability, or intracellular distribution, palmitoylation of the immune molecule contributes to STAT1/2 sensitization and nuclear translocation to facilitate the gene transcription induced by IFN-α (Figure 8).

Similar to JAK1, the roles of STAT3 palmitoylation and associated molecular mechanisms have been investigated by different groups. Zhang et al. showed that STAT3 can be palmitoylated at Cys108. ZDHHC3 and ZDHHC7 are capable of palmitoylating STAT3, leading to its recruitment to the cell membrane and phosphorylation. Conversely. APT2 depalmitoylates phosphorylated STAT3 to enhance its translocation to the nucleus. STAT3 is a critical Th17 cell differentiation stimulator. The palmitoylation–depalmitoylation cycle of STAT3 contributes to the regulation of Th17 cell differentiation.^105^ In particular, over-activation of Th17 cells is responsible for the development of inflammatory bowel disease (IBD). Knockout of STAT3’s palmitoylase ZDHHC7 relieves IBD symptoms in mouse models (Figure 8). In addition, long-chain fatty acids (LCFAs), are significant triggering factors for IBD. A study from Wei et al. showed that PA, a representative LCFA, can enter intestinal epithelial cells via CD36 to regulate the STAT3 palmitoylation cycle.^106^ Inhibiting the cycle using the palmitoylation inhibitor 2-BP, or knockdown of ZDHHC7, can alleviate inflammation in the mouse model induced by PA. In HCC cells, palmitoylation of STAT3 was also increased to enhance its activity.^107^ Inhibiting ZDHHC7 decreased the palmitoylation of STAT3 and reduced its downstream gene HIF1α, indicating that STAT3 palmitoylation induced by ZDHHC7 contributes to HCC malignancy. In addition to ZDHHC7, Ma et al. found that, in oligodendrocytes, ZDHHC5 can palmitoylate STAT3 at Cys687 and Cys712 in the C-transactivation domain, contributing to its phosphorylation, activation, and recruitment of STAT3 to chromatin, facilitating oligodendrocyte development.^108^ Furthermore, ZDHHC5-dependent palmitoylation promotes the interaction of STAT3 with JAK1 and JAK2.

Increasing evidence indicates that STAT3 activation can also be controlled by other palmitoylated proteins. For example, in spinal astrocytes, GFAP can be palmitoylcated by ZDHHC5, ZDHHC12, ZDHHC21, and ZDHHC23. Furthermore, GFAP palmitoylcation at Cys291 mediated by ZDHHC23 increased the secretion of the cytokines CXCL-10, IL-6, and GM-CSF, which further induced STAT3 activation.^109^ In glioblastoma (GBM) stem cells, palmitoylation Glycogen synthase kinase 3β (GSK3β) at Cys14 mediated by ZDHHC4 can activate STAT3 signaling to improve the stemness of temozolomide (TMZ)-resistant GBM.^110^ In dorsal root ganglion axons, ZDHHC5 and ZDHHC8 can palmitoylate Gp130 to increase its surface expression and then activate STAT3 (Figure 8).^111^ To dates, the palmitoylation sites in Gp130 mediated by ZDHHCs have not been investigated. ZDHHC15 promotes STAT3 signaling activation in glioma.^112^ However, it is unclear whether ZDHHC15-mediated activation relies on STAT3 palmitoylation.

## Palmitoylation is a potential treatment target in PRR-related diseases

For the treatment of diseases with protein palmitoylation, targeting this PTM is based on three directions: targeting substrate palmitoylation, inhibiting ZDHHC enzymes, and preventing depalmitoylation.^26^ Owing to the significance of palmitoylation activating PRR-signaling, targeting the palmitoylation of the core molecules in PRRs with specific pharmaceuticals is a vital treatment strategy for PRR-associated diseases. A recent study showed that caffeine, a representative ingredient of coffee, can inhibit the development of nonalcoholic steatohepatitis (NASH). Moreover, work using human liver cell models suggested that the caffeine-mediated suppression of NASH is linked to a reduction in Myd88 palmitoylation, which further weakens NF-κB-dependent inflammatory reactions by decreasing the production of TNF-α and IL-6.^31^

Recently, small molecules targeting STING palmitoylation have been identified and developed. For instance, 4-octyl itaconate (4-OI), a derivative of itaconate, can inhibit cGAS-STING activation by alkylating STING at Cys91 to block its palmitoylation and oligomerization.^39^ Furthermore, 4-OI suppressed antiviral immune responses to the autoimmune inflammation mediated by cGAS-STING signaling in mouse bone marrow-derived macrophages (BMDMs) and Raw264.7 cells. During HSV-2 infection, nitro-fatty acids are formed, and nitro-fatty acids are capable of nitroalkylating human STING at Cys88 and Cys91 to inhibit its palmitoylation and signaling activation (Table 1),^14^ suggesting that nitro-fatty acids are endogenous inhibitors of STING signaling and can be considered antagonists for the treatment of STING-dependent inflammatory diseases.

**Table 1:** The molecules as potential pharmaceuticals targeting the palmitoylation of specific immune proteins in PRRs

Molecule	Target protein	Target palmitoylation sites in protein	Effect on Target protein palmitoylation	The species origin of the target protein	References
Caffeine	Myd88	Unknown	Inhibition	Human	Tan et al.^31^
4-OI	STING	Cys91	Inhibition	Mouse	Su et al.^39^
Nitro-fatty acids	STING	Cys91	Inhibition	Human	Hansen et al.^14^
C-176	STING	Cys91	Inhibition	Mouse	Haag et al.^29^
C-178	STING	Cys91	Inhibition	Mouse	Haag et al.^29^
C-170	STING	Cys91	Inhibition	Human/Mouse	Haag et al.^29^
C-171	STING	Cys91	Inhibition	Human/Mouse	Haag et al.^29^
H-151	STING	Cys91	Inhibition	Human	Haag et al.^29^
excB	STING	Cys91	Inhibition	Mouse	Hsiao et al.^113^
PAO	STING	Cys88/Cys91	Activation	Mouse	Matsumoto et al.^114^
Disulfiram	GSDMD-NT	Cys192	Inhibition	Mouse	Zhuang et al.^100^
Disulfiram	NLRP3	Cys126	Inhibition	Human/Mouse	Xu et al.^69^
Vaccarin	NLRP3	Cys844	Activation	Mouse	Wang et al.^36^ Zhu et al.^115^
2-BP	TLR9	Cys258/Cys265	Inhibition	Human/Mouse	Ni et al.^10^
2-BP	cGAS	Cys404/Cys405	Inhibition	Human	Fan et al.^13^
2-BP	STING	Cys88/Cys91	Inhibition	Human	Zhu et al.^64^
2-BP	MAVS	Cys508	Inhibition	Mouse	Liu et al.^55^
2-BP	NLRP3	Cys419	Inhibition	Mouse	Hu et al.^68^
CMA	GSDMD	Cys191	Inhibition	Human	Liu et al.^94^
ML349	MAVS	Cys46/Cys79	Activation	Human/Mouse	Bu et al.^54^
HDSF	TLR9	Cys258/Cys265	Activation	Human/Mouse	Ni et al.^10^

C-176 and C-178, two nitrofuran derivatives, have been demonstrated to target STING at Cys91 in mice directly but not in humans to repress the palmitoylation and signaling of STING.^29^ C-170 and C-171, two derivatives of C-176 and C-178, can act as both human and mouse STING antagonists at Cys91. H-151, a more advanced STING antagonist, has also been shown to effectively block the palmitoylation of STING on Cys91 in humans (Table 1). Hsiao et al. also discovered that excavatolide B (excB), a natural product in the cultured soft coral Briareum stechei, could target mouse STING at Cys91 and inhibit STING palmitoylation and signaling in mouse RAW 264.7 cells.^113^ Whether excB has an inhibitory effect on human STING warrants further investigation.

Phenylarsine oxide (PAO) (Table 1), a non-nucleotide agonist targeting Cys88 and Cys291 of mouse STING, was discovered by Matsumoto et al.^114^ Given that STING activation mediated by PAO is dependent on the palmitoylation of immune molecules and that STING activation mediated by PAO does not require stimulation mediated by cGAS or cGAMP, it is reasonable to speculate that the palmitoylated Cys residues in STING are ideal targets for the development of STING agonists.

A previous study shows that the palmitoylation of GSDMD-NT at Cys192 is antagonized by disulfiram (Table 1), a drug used for treating alcoholism in the clinic, to reduce myocardial pyroptosis in acute myocardial infarction (AMI) mice.^100^ Interestingly, disulfiram has also been demonstrated to reduce NLRP3 palmitoylation mediated by ZDHCC5 at Cys126 to inhibit inflammasome activation in human macrophages and mouse models of sepsis and peritonitis.^69^ In addition, vaccarin, a flavonoid component, can induce NLRP3 palmitoylation mediated by ZDHHC12 to inactivate the NLRP3 inflammasome and alleviate septic cardiomyopathy.^115^

Protein palmitoylation is a reversible process that is modulated by palmitoylases and depalmitoylases.^8^ ZDHHCs are the only identified palmitoylases. Targeting ZDHHCs is an attractive disease treatment strategy. However, many proteins in PRR-signaling could be palmitoylated by more than one ZDHHC enzyme. Therefore, the suppression of a single ZDHHC does not fully restrict the palmitoylation of substrate proteins. One potential solution to this problem is the development of pan-ZDHHC inhibitors that block the biological functions of several enzymes required for palmitoylation of a single target.^26^ Until now, only a few pan-ZDHHC inhibitors, including compound V, cerulenin, tunicamycin, cyano-myracrylamide (CMA),^94^ and 2-BP, have been reported. Among these inhibitors, 2-BP is under investigation in preclinical studies to validate whether pan-ZDHHC inhibition be used to treat diseases.^26^ Especially, based on cell and mouse models, it has been shown that treatment with 2-BP effectively inhibits TLR9 palmitoylation at Cys258 and Cys265 and suppresses the production of IFNα in SLE.^10^ Another investigation indicated that 2-BP decreased cellular DNA sensing mediated by cGAS palmitoylation at Cys404/405 in human MDA-MB-231 cancer cells and subsequently impaired IRF3 signaling activation after stimulation with HT-DNA, dsDNA, and HSV-1 infection.^13^ In human RCC cells, inhibiting STING palmitoylases at Csy88 and Cys91 with 2-BP significantly reduces cancer cell growth (Table 1).^64^ Lin et al. showed that 2-BP could inhibit MAVS palmitoylation at Cys508 to reduce the aggregation of this molecule on the mitochondrial outer membrane and subsequently block antiviral innate immunity.^55^ Relying on inflammatory bowel disease mouse models, 2-BP treatment can attenuate weight loss, rescue pathological changes, and improve the survival rate by inhibiting NLRP3 palmitoylation at Cys419.^68^ In addition, CMA inhibits human GSDMD palmitoylation at Cys191 to suppress its localization to the membrane and dampen pyroptosis and IL-1β secretion.^94^

In addition to inhibiting protein palmitoylation, it may be more effective in preventing depalmitoylation for treating PRR-associated diseases. Depalmitoylating enzymes are highly druggable targets, and different inhibitors, including palmostatin B (Palm B), hexadecylsulfonyl fluoride (HDSF),^10^ ML348, and ML349, have been developed to target depalmitoylating enzymes.^26^ In particular, Guo et al. found that targeting APT2 with its inhibitor ML349 can benefit MAVS palmitoylation at Cys46 and Cys79 and promote antiviral innate responses in human cells and mice.^54^ Ni et al. showed that HDSF, a depalmitoylating enzyme PPT1 inhibitor (Table 1), is capable of suppressing IFNα production by promoting palmitoylation of TLR9 at Cys258 and Cys265 in SLE.^10^ However, whether these depalmitoylase inhibitors could be used in clinical to treat PRR-related illness needs to be explored.

## Conclusions and future perspectives

As important innate immune receptors, PRRs modulate a variety of physiological and pathological processes. Because PRRs are dysregulated in viral infection, autoimmune diseases, and tumors and have significant effects on the occurrence and progression of these disorders,^3^ the clinical implications of potent drugs, including agonists and antagonists targeting PRRs, for treating these diseases are being assessed clinically and preclinically.^3^^,^^116^ The molecular mechanisms linked to the modulation of PRR-signaling are complicated. Currently, much attention has been given to PTMs, which are critical for the initiation, progression, and outcome of various diseases associated with PRR-signaling.^7^ Until now, more than 600 types of PTMs have been identified.^20^ However, the role of a large number of PTMs in modulating PRR-signaling still needs to be better understood. Therefore, to better target PRRs in clinical treatment, a more thorough understanding of the effects of different classes of PTMs on the regulation of PRRs is urgently needed.

In recent years, significant progress has shown that palmitoylation is essential for the regulation of the immune response.^9^^,^^22^ In particular, our reviewed studies demonstrated that palmitoylation could control the activation of various classes of PRRs, including TLRs, RLRs, cytoplasmic DNA sensors, NLRs, and their downstream NF-κB, MAPK, inflammasome, and JAK-STAT signaling pathways, both directly and indirectly. Furthermore, the available evidence suggests that targeting palmitoylation is beneficial for controlling diseases caused by dysregulated PPRs. Therefore, further identification of the diseases with the dysregulation of PRRs and their signaling pathways controlled by palmitoylation may aid in the development of effective intervention strategies.

To date, although we have learned the vital role of palmitoylation in the regulation of PRR-signaling, many crucial issues remain. (1) We have little information on how palmitoylases and depalmitoylases are modulated to mediate the palmitoylation and depalmitoylation of different molecules in PRR-signaling. (2) Although the palmitoylation of many molecules in PRRs has been identified, the effect of palmitoylation on the modulation of these molecular functions is still not fully evaluated. (3) Using cell and mouse models, the present studies mainly focused on investigating the effect of palmitoylation on proteins in PRR-signaling *in vitro* and *in vivo*, whether palmitoylated PRR-signaling associated molecules could be used for clinical diagnosis or serve as parameters predicting the prognosis of different diseases are still undefined. (4) Current evidence indicates that different palmitoylation sites in the same molecule have diverse effects.^15^^,^^36^^,^^72^ If many palmitoylation sites are identified in immune molecules involved in PRR-signaling, the exact roles of individual palmitoylation sites should be investigated in detail in future studies. (5) Potential pharmaceuticals targeting different molecules with palmitoylation in PRRs have been discovered (Table 1), and whether these pharmaceuticals could be used for the treatment of patients with PRR-associated diseases should be estimated based on preclinical and clinical trials.

## Acknowledgments

This study was supported by the Qing Lan Project of Jiangsu Province, the Natural Science Foundation of the Jiangsu Higher Education Institutions (21KJA310004), the 10.13039/501100004608Natural Science Foundation of Jiangsu Province (BK20211347), and a project funded by the 10.13039/501100012246Priority Academic Program Development of Jiangsu Higher Education Institutions (PAPD). The figures used in the paper were created with BioRender (https://biorender.com/).

## Author contributions

K.Z., R.T., and F.K. conceived and designed the review; X.L., X.H., and F.K. searched the literature and collected the data; X.L., X.H., H.Y., and F.K. wrote the paper and made the illustrations.

## Declaration of interests

The authors declare no competing interests.

## References

[1] Ma M., Jiang W., Zhou R. (2024). DAMPs and DAMP-sensing receptors in inflammation and diseases. Immunity.

[2] Gong T., Liu L., Jiang W., Zhou R. (2020). DAMP-sensing receptors in sterile inflammation and inflammatory diseases. Nat. Rev. Immunol..

[3] Li D., Wu M. (2021). Pattern recognition receptors in health and diseases. Signal Transduct. Targeted Ther..

[4] Kong F., You H., Zheng K., Tang R., Zheng C. (2021). The crosstalk between pattern-recognition receptor signaling and calcium signaling. Int. J. Biol. Macromol..

[5] You H., Qin S., Zhang F., Hu W., Li X., Liu D., Kong F., Pan X., Zheng K., Tang R. (2022). Regulation of Pattern-Recognition Receptor Signaling by HBX During Hepatitis B Virus Infection. Front. Immunol..

[6] Lee M.S., Kim Y.J. (2007). Signaling pathways downstream of pattern-recognition receptors and their cross talk. Annu. Rev. Biochem..

[7] Liu J., Qian C., Cao X. (2016). Post-Translational Modification Control of Innate Immunity. Immunity.

[8] S Mesquita F., Abrami L., Linder M.E., Bamji S.X., Dickinson B.C., van der Goot F.G. (2024). Mechanisms and functions of protein S-acylation. Nat. Rev. Mol. Cell Biol..

[9] Lin H. (2021). Protein cysteine palmitoylation in immunity and inflammation. FEBS J..

[10] Ni H., Wang Y., Yao K., Wang L., Huang J., Xiao Y., Chen H., Liu B., Yang C.Y., Zhao J. (2024). Cyclical palmitoylation regulates TLR9 signalling and systemic autoimmunity in mice. Nat. Commun..

[11] Chesarino N.M., Hach J.C., Chen J.L., Zaro B.W., Rajaram M.V., Turner J., Schlesinger L.S., Pratt M.R., Hang H.C., Yount J.S. (2014). Chemoproteomics reveals Toll-like receptor fatty acylation. BMC Biol..

[12] Zhang G., Jiang P., Tang W., Wang Y., Qiu F., An J., Zheng Y., Wu D., Zhou J., Neculai D. (2023). CPT1A induction following epigenetic perturbation promotes MAVS palmitoylation and activation to potentiate antitumor immunity. Mol. Cell.

[13] Fan Y., Gao Y., Nie L., Hou T., Dan W., Wang Z., Liu T., Wei Y., Wang Y., Liu B. (2023). Targeting LYPLAL1-mediated cGAS depalmitoylation enhances the response to anti-tumor immunotherapy. Mol. Cell.

[14] Hansen A.L., Buchan G.J., Rühl M., Mukai K., Salvatore S.R., Ogawa E., Andersen S.D., Iversen M.B., Thielke A.L., Gunderstofte C. (2018). Nitro-fatty acids are formed in response to virus infection and are potent inhibitors of STING palmitoylation and signaling. Proc. Natl. Acad. Sci. USA.

[15] Yu T., Hou D., Zhao J., Lu X., Greentree W.K., Zhao Q., Yang M., Conde D.G., Linder M.E., Lin H. (2024). NLRP3 Cys126 palmitoylation by ZDHHC7 promotes inflammasome activation. Cell Rep..

[16] Jiang L., Wang Z., Xu T., Zhang L. (2024). When pyro(ptosis) meets palm(itoylation). Cytokine Growth Factor Rev..

[17] Gao J., Li W., Zhang Z., Gao W., Kong E. (2022). Proteome-wide identification of palmitoylated proteins in mouse testis. Reprod. Sci..

[18] Niu J., Holland S.M., Ketschek A., Collura K.M., Hesketh N.L., Hayashi T., Gallo G., Thomas G.M. (2022). Palmitoylation couples the kinases DLK and JNK3 to facilitate prodegenerative axon-to-soma signaling. Sci. Signal..

[19] Ren W., Jhala U.S., Du K. (2013). Proteomic analysis of protein palmitoylation in adipocytes. Adipocyte.

[20] Wu X., Xu M., Geng M., Chen S., Little P.J., Xu S., Weng J. (2023). Targeting protein modifications in metabolic diseases: molecular mechanisms and targeted therapies. Signal Transduct. Targeted Ther..

[21] Jin J., Zhi X., Wang X., Meng D. (2021). Protein palmitoylation and its pathophysiological relevance. J. Cell. Physiol..

[22] Cai J., Cui J., Wang L. (2023). S-palmitoylation regulates innate immune signaling pathways: molecular mechanisms and targeted therapies. Eur. J. Immunol..

[23] Yi L., Zheng C. (2021). The emerging roles of ZDHHCs-mediated protein palmitoylation in the antiviral innate immune responses. Crit. Rev. Microbiol..

[24] Shi X., Li X., Xu Z., Shen L., Ding Y., Chen S., Mao L., Liu W., Xu J. (2022). ABHD16A Negatively Regulates the Palmitoylation and Antiviral Function of IFITM Proteins. mBio.

[25] Lin X., Shi Y., Zhan Y., Xing Y., Li Y., Zhou Z., Chen G. (2023). Advances of Protein Palmitoylation in Tumor Cell Deaths. Cancers.

[26] Li M., Zhang L., Chen C.W. (2023). Diverse Roles of Protein Palmitoylation in Cancer Progression, Immunity, Stemness, and Beyond. Cells.

[27] Li X., Shen L., Xu Z., Liu W., Li A., Xu J. (2022). Protein Palmitoylation Modification During Viral Infection and Detection Methods of Palmitoylated Proteins. Front. Cell. Infect. Microbiol..

[28] Mukai K., Konno H., Akiba T., Uemura T., Waguri S., Kobayashi T., Barber G.N., Arai H., Taguchi T. (2016). Activation of STING requires palmitoylation at the Golgi. Nat. Commun..

[29] Haag S.M., Gulen M.F., Reymond L., Gibelin A., Abrami L., Decout A., Heymann M., van der Goot F.G., Turcatti G., Behrendt R., Ablasser A. (2018). Targeting STING with covalent small-molecule inhibitors. Nature.

[30] Chow A., Zhou W., Liu L., Fong M.Y., Champer J., Van Haute D., Chin A.R., Ren X., Gugiu B.G., Meng Z. (2014). Macrophage immunomodulation by breast cancer-derived exosomes requires Toll-like receptor 2-mediated activation of NF-κB. Sci. Rep..

[31] Tan X., Sun Y., Chen L., Hu J., Meng Y., Yuan M., Wang Q., Li S., Zheng G., Qiu Z. (2022). Caffeine ameliorates AKT-driven nonalcoholic steatohepatitis by suppressing de novo lipogenesis and MyD88 palmitoylation. J. Agric. Food Chem..

[32] Kim Y.C., Lee S.E., Kim S.K., Jang H.D., Hwang I., Jin S., Hong E.B., Jang K.S., Kim H.S. (2019). Toll-like receptor mediated inflammation requires FASN-dependent MYD88 palmitoylation. Nat. Chem. Biol..

[33] Hao S., Zheng X., Zhu Y., Yao Y., Li S., Xu Y., Feng W.H. (2023). African swine fever virus QP383R dampens type I interferon production by promoting cGAS palmitoylation. Front. Immunol..

[34] Shi C., Yang X., Liu Y., Li H., Chu H., Li G., Yin H. (2022). ZDHHC18 negatively regulates cGAS-mediated innate immunity through palmitoylation. EMBO J..

[35] Yang M., Jiang H., Ding C., Zhang L., Ding N., Li G., Zhang F., Wang J., Deng L., Liu J., Xu Y. (2023). STING activation in platelets aggravates septic thrombosis by enhancing platelet activation and granule secretion. Immunity.

[36] Wang L., Cai J., Zhao X., Ma L., Zeng P., Zhou L., Liu Y., Yang S., Cai Z., Zhang S. (2023). Palmitoylation prevents sustained inflammation by limiting NLRP3 inflammasome activation through chaperone-mediated autophagy. Mol. Cell.

[37] Dixon C.L., Martin N.R., Niphakis M.J., Cravatt B.F., Fairn G.D. (2023). Attenuating ABHD17 enhances S-palmitoylation, membrane localization and signal transduction of NOD2 and Crohn’s disease-associated variants. bioRxiv.

[38] Zhou L., He X., Wang L., Wei P., Cai Z., Zhang S., Jin S., Zeng H., Cui J. (2022). Palmitoylation restricts SQSTM1/p62-mediated autophagic degradation of NOD2 to modulate inflammation. Cell Death Differ..

[39] Su C., Cheng T., Huang J., Zhang T., Yin H. (2023). 4-Octyl itaconate restricts STING activation by blocking its palmitoylation. Cell Rep..

[40] Zheng S., Que X., Wang S., Zhou Q., Xing X., Chen L., Hou C., Ma J., An P., Peng Y. (2023). ZDHHC5-mediated NLRP3 palmitoylation promotes NLRP3-NEK7 interaction and inflammasome activation. Mol. Cell.

[41] Kawai T., Ikegawa M., Ori D., Akira S. (2024). Decoding Toll-like receptors: Recent insights and perspectives in innate immunity. Immunity.

[42] Tohumeken S., Baur R., Böttcher M., Stoll A., Loschinski R., Panagiotidis K., Braun M., Saul D., Völkl S., Baur A.S. (2020). Palmitoylated Proteins on AML-Derived Extracellular Vesicles Promote Myeloid-Derived Suppressor Cell Differentiation via TLR2/Akt/mTOR Signaling. Cancer Res..

[43] Nguyen M.T.A., Favelyukis S., Nguyen A.K., Reichart D., Scott P.A., Jenn A., Liu-Bryan R., Glass C.K., Neels J.G., Olefsky J.M. (2007). A subpopulation of macrophages infiltrates hypertrophic adipose tissue and is activated by free fatty acids via Toll-like receptors 2 and 4 and JNK-dependent pathways. J. Biol. Chem..

[44] Shi H., Kokoeva M.V., Inouye K., Tzameli I., Yin H., Flier J.S. (2006). TLR4 links innate immunity and fatty acid-induced insulin resistance. J. Clin. Invest..

[45] Lancaster G.I., Langley K.G., Berglund N.A., Kammoun H.L., Reibe S., Estevez E., Weir J., Mellett N.A., Pernes G., Conway J.R.W. (2018). Evidence that TLR4 Is Not a Receptor for Saturated Fatty Acids but Mediates Lipid-Induced Inflammation by Reprogramming Macrophage Metabolism. Cell Metabol..

[46] Borzęcka-Solarz K., Dembinska J., Hromada-Judycka A., Traczyk G., Ciesielska A., Ziemlinska E., Swiatkowska A., Kwiatkowska K. (2017). Association of Lyn kinase with membrane rafts determines its negative influence on LPS-induced signaling. Mol. Biol. Cell.

[47] Kim K.S., Kim J.S., Park J.Y., Suh Y.H., Jou I., Joe E.H., Park S.M. (2013). DJ-1 associates with lipid rafts by palmitoylation and regulates lipid rafts-dependent endocytosis in astrocytes. Hum. Mol. Genet..

[48] Tao L., Liu Y., Fan G., Zhang H., Zong Y., Yang X. (2023). GRK6 palmitoylation increasing its membrance translocation promotes LPS-induced inflammation by PI3K/AKT pathway in kuppfer cells. Int. Immunopharm..

[49] Sobocińska J., Roszczenko-Jasinska P., Zareba-Koziol M., Hromada-Judycka A., Matveichuk O.V., Traczyk G., Lukasiuk K., Kwiatkowska K. (2018). Lipopolysaccharide Upregulates Palmitoylated Enzymes of the Phosphatidylinositol Cycle: An Insight from Proteomic Studies. Mol. Cell. Proteomics.

[50] Zhou B., Yang W., Li W., He L., Lu L., Zhang L., Liu Z., Wang Y., Chao T., Huang R. (2020). Zdhhc2 Is Essential for Plasmacytoid Dendritic Cells Mediated Inflammatory Response in Psoriasis. Front. Immunol..

[51] Lu J., Zhong X., Guo C., Tang L., Yu N., Peng C., Ding Y., Bao X., Zhou J., Shi Y. (2023). TLR7-MyD88-DC-CXCL16 axis results neutrophil activation to elicit inflammatory response in pustular psoriasis. Cell Death Dis..

[52] Liu E., Sun J., Yang J., Li L., Yang Q., Zeng J., Zhang J., Chen D., Sun Q. (2021). ZDHHC11 positively regulates NF-κB activation by enhancing TRAF6 oligomerization. Front. Cell Dev. Biol..

[53] Yoneyama M., Kato H., Fujita T. (2024). Physiological functions of RIG-I-like receptors. Immunity.

[54] Bu L., Wang H., Zhang S., Zhang Y., Liu M., Zhang Z., Wu X., Jiang Q., Wang L., Xie W. (2024). Targeting APT2 improves MAVS palmitoylation and antiviral innate immunity. Mol. Cell.

[55] Liu Y., Hou D., Chen W., Lu X., Komaniecki G.P., Xu Y., Yu T., Zhang S.M., Linder M.E., Lin H. (2024). MAVS Cys508 palmitoylation promotes its aggregation on the mitochondrial outer membrane and antiviral innate immunity. Proc. Natl. Acad. Sci. USA.

[56] Yang S., Harding A.T., Sweeney C., Miao D., Swan G., Zhou C., Jiang Z., Fitzgerald K.A., Hammer G., Bergo M.O. (2019). Control of antiviral innate immune response by protein geranylgeranylation. Sci. Adv..

[57] Chin E.N., Sulpizio A., Lairson L.L. (2023). Targeting STING to promote antitumor immunity. Trends Cell Biol..

[58] Dvorkin S., Cambier S., Volkman H.E., Stetson D.B. (2024). New frontiers in the cGAS-STING intracellular DNA-sensing pathway. Immunity.

[59] Guo B., Chen J.H., Zhang J.H., Fang Y., Liu X.J., Zhang J., Zhu H.Q., Zhan L. (2023). Pattern-recognition receptors in endometriosis: A narrative review. Front. Immunol..

[60] Kang J., Wu J., Liu Q., Jiang H., Li W., Li Y., Li X., Ni C., Wu L., Liu M. (2024). FASN regulates STING palmitoylation via malonyl-CoA in macrophages to alleviate sepsis-induced liver injury. Biochim. Biophys. Acta, Mol. Basis Dis..

[61] Gao L., Tian T., Xiong T., Zhang X., Wang N., Liu L., Shi Y., Liu Q., Lu D., Luo P. (2024). Type VII secretion system extracellular protein B targets STING to evade host anti--Staphylococcus aureus immunity. Proc. Natl. Acad. Sci. USA.

[62] Kemmoku H., Takahashi K., Mukai K., Mori T., Hirosawa K.M., Kiku F., Uchida Y., Kuchitsu Y., Nishioka Y., Sawa M. (2024). Single-molecule localization microscopy reveals STING clustering at the trans-Golgi network through palmitoylation-dependent accumulation of cholesterol. Nat. Commun..

[63] Dogrammatzis C., Saud R., Waisner H., Lasnier S., Suma S.M., Grieshaber B., Kalamvoki M. (2024). Tracing the STING exocytosis pathway during herpes viruses infection. mBio.

[64] Zhu Z., Zhou X., Du H., Cloer E.W., Zhang J., Mei L., Wang Y., Tan X., Hepperla A.J., Simon J.M. (2023). STING Suppresses Mitochondrial VDAC2 to Govern RCC Growth Independent of Innate Immunity. Adv. Sci..

[65] Sundaram B., Tweedell R.E., Prasanth Kumar S., Kanneganti T.D. (2024). The NLR family of innate immune and cell death sensors. Immunity.

[66] Madahar S.S., Gideon A., Abdul-Sater A.A. (2024). Nod-like receptors in inflammatory arthritis. Biomed. J..

[67] O’Keefe M.E., Dubyak G.R., Abbott D.W. (2024). Post-translational control of NLRP3 inflammasome signaling. J. Biol. Chem..

[68] Hu D., Li Y., Wang X., Zou H., Li Z., Chen W., Meng Y., Wang Y., Li Q., Liao F. (2024). Palmitoylation of NLRP3 Modulates Inflammasome Activation and Inflammatory Bowel Disease Development. J. Immunol..

[69] Xu J., Pickard J.M., Núñez G. (2024). FDA-approved disulfiram inhibits the NLRP3 inflammasome by regulating NLRP3 palmitoylation. Cell Rep..

[70] Nie L., Fei C., Fan Y., Dang F., Zhao Z., Zhu T., Wu X., Dai T., Balasubramanian A., Pan J. (2024). Consecutive palmitoylation and phosphorylation orchestrates NLRP3 membrane trafficking and inflammasome activation. Mol. Cell.

[71] Lv D., Cao X., Zhong L., Dong Y., Xu Z., Rong Y., Xu H., Wang Z., Yang H., Yin R. (2023). Targeting phenylpyruvate restrains excessive NLRP3 inflammasome activation and pathological inflammation in diabetic wound healing. Cell Rep. Med..

[72] Leishman S., Aljadeed N.M., Qian L., Cockcroft S., Behmoaras J., Anand P.K. (2024). Fatty acid synthesis promotes inflammasome activation through NLRP3 palmitoylation. Cell Rep..

[73] Yang S., Li M., Lian G., Wu Y., Cui J., Wang L. (2024). ABHD8 antagonizes inflammation by facilitating chaperone-mediated autophagy-mediated degradation of NLRP3. Autophagy.

[74] Lu Y., Zheng Y., Coyaud É., Zhang C., Selvabaskaran A., Yu Y., Xu Z., Weng X., Chen J.S., Meng Y. (2019). Palmitoylation of NOD1 and NOD2 is required for bacterial sensing. Science.

[75] Wang K., Huang H., Zhan Q., Ding H., Li Y. (2024). Toll-like receptors in health and disease. MedComm.

[76] Guo Q., Jin Y., Chen X., Ye X., Shen X., Lin M., Zeng C., Zhou T., Zhang J. (2024). NF-κB in biology and targeted therapy: new insights and translational implications. Signal Transduct. Targeted Ther..

[77] Aqdas M., Sung M.H. (2023). NF-kappaB dynamics in the language of immune cells. Trends Immunol..

[78] Li J.K., Rao Y.Q., Koh S.K., Zhao P., Zhou L., Li J. (2022). Proteomic analysis of s-acylated proteins in human retinal pigment epithelial cells and the role of palmitoylation of Niemann-Pick type C1 protein in cholesterol transport. Front. Aging Neurosci..

[79] Zingler P., Särchen V., Glatter T., Caning L., Saggau C., Kathayat R.S., Dickinson B.C., Adam D., Schneider-Brachert W., Schütze S., Fritsch J. (2019). Palmitoylation is required for TNF-R1 signaling. Cell Commun. Signal..

[80] Bekhouche B., Tourville A., Ravichandran Y., Tacine R., Abrami L., Dussiot M., Khau-Dancasius A., Boccara O., Khirat M., Mangeney M. (2020). A toxic palmitoylation of Cdc42 enhances NF-κB signaling and drives a severe autoinflammatory syndrome. J. Allergy Clin. Immunol..

[81] Guo H.Z., Feng R.X., Zhang Y.J., Yu Y.H., Lu W., Liu J.J., Yang S.X., Zhao C., Zhang Z.L., Yu S.H. (2024). A CD36-dependent non-canonical lipid metabolism program promotes immune escape and resistance to hypomethylating agent therapy in AML. Cell Rep. Med..

[82] Braicu C., Buse M., Busuioc C., Drula R., Gulei D., Raduly L., Rusu A., Irimie A., Atanasov A.G., Slaby O. (2019). A Comprehensive Review on MAPK: A Promising Therapeutic Target in Cancer. Cancers.

[83] Azizi S.A., Qiu T., Brookes N.E., Dickinson B.C. (2023). Regulation of ERK2 activity by dynamic S-acylation. Cell Rep..

[84] Botham A., Guo X., Xiao Y.P., Morice A.H., Compton S.J., Sadofsky L.R. (2011). Palmitoylation of human proteinase-activated receptor-2 differentially regulates receptor-triggered ERK1/2 activation, calcium signalling and endocytosis. Biochem. J..

[85] Ren J.G., Xing B., Lv K., O'Keefe R.A., Wu M., Wang R., Bauer K.M., Ghazaryan A., Burslem G.M., Zhang J. (2023). RAB27B controls palmitoylation-dependent NRAS trafficking and signaling in myeloid leukemia. J. Clin. Invest..

[86] Zhang F., Fu Y., Wang J., Lang L., Liang S., Zhang S., Wang L., Gao P., Shu G., Zhu C. (2024). Conjugated linoleic acid (CLA) reduces intestinal fatty acid uptake and chylomicron formation in HFD-fed mice associated with the inhibition of DHHC7-mediated CD36 palmitoylation and the downstream ERK pathway. Food Funct..

[87] Yang G., Liu Y., Yang K., Liu R., Zhu S., Coquinco A., Wen W., Kojic L., Jia W., Cynader M. (2012). Isoform-specific palmitoylation of JNK regulates axonal development. Cell Death Differ..

[88] Hao J.W., Wang J., Guo H., Zhao Y.Y., Sun H.H., Li Y.F., Lai X.Y., Zhao N., Wang X., Xie C. (2020). CD36 facilitates fatty acid uptake by dynamic palmitoylation-regulated endocytosis. Nat. Commun..

[89] Zhao L., Zhang C., Luo X., Wang P., Zhou W., Zhong S., Xie Y., Jiang Y., Yang P., Tang R. (2018). CD36 palmitoylation disrupts free fatty acid metabolism and promotes tissue inflammation in non-alcoholic steatohepatitis. J. Hepatol..

[90] Caiazza F., Galluzzo P., Lorenzetti S., Marino M. (2007). 17Beta-estradiol induces ERbeta up-regulation via p38/MAPK activation in colon cancer cells. Biochem. Biophys. Res. Commun..

[91] Veluthakal R., Kumar B., Mohammad G., Kowluru A., Kowluru R.A. (2015). Tiam1-Rac1 Axis Promotes Activation of p38 MAP Kinase in the Development of Diabetic Retinopathy: Evidence for a Requisite Role for Protein Palmitoylation. Cell. Physiol. Biochem..

[92] Xu J., Núñez G. (2023). The NLRP3 inflammasome: activation and regulation. Trends Biochem. Sci..

[93] Stine L., Humphries F. (2024). Gasdermin D palmitoylation: to cleave or not to cleave?. Trends Immunol..

[94] Liu Z., Li S., Wang C., Vidmar K.J., Bracey S., Li L., Willard B., Miyagi M., Lan T., Dickinson B.C. (2024). Palmitoylation at a conserved cysteine residue facilitates gasdermin D-mediated pyroptosis and cytokine release. Proc. Natl. Acad. Sci. USA.

[95] Margheritis E., Kappelhoff S., Danial J., Gehle N., Kohl W., Kurre R., González Montoro A., Cosentino K. (2024). Gasdermin D cysteine residues synergistically control its palmitoylation-mediated membrane targeting and assembly. EMBO J..

[96] Zhang N., Zhang J., Yang Y., Shan H., Hou S., Fang H., Ma M., Chen Z., Tan L., Xu D. (2024). A palmitoylation-depalmitoylation relay spatiotemporally controls GSDMD activation in pyroptosis. Nat. Cell Biol..

[97] Balasubramanian A., Hsu A.Y., Ghimire L., Tahir M., Devant P., Fontana P., Du G., Liu X., Fabin D., Kambara H. (2024). The palmitoylation of gasdermin D directs its membrane translocation and pore formation during pyroptosis. Sci. Immunol..

[98] Sun Z., Hornung V. (2024). A critical role for palmitoylation in pyroptosis. Mol. Cell.

[99] Du G., Healy L.B., David L., Walker C., El-Baba T.J., Lutomski C.A., Goh B., Gu B., Pi X., Devant P. (2024). ROS-dependent S-palmitoylation activates cleaved and intact gasdermin D. Nature.

[100] Zhuang Z., Gu J., Li B.O., Yang L. (2024). Inhibition of gasdermin D palmitoylation by disulfiram is crucial for the treatment of myocardial infarction. Transl. Res..

[101] Zhou Y., Yue S., Li L., Zhang J., Chen L., Chen J. (2024). SMPDL3B is palmitoylated and stabilized by ZDHHC5, and its silencing aggravates diabetic retinopathy of db/db mice: Activation of NLRP3/NF-κB pathway. Cell. Signal..

[102] Hu X., Li J., Fu M., Zhao X., Wang W. (2021). The JAK/STAT signaling pathway: from bench to clinic. Signal Transduct. Targeted Ther..

[103] Hernandez L.M., Montersino A., Niu J., Guo S., Faezov B., Sanders S.S., Dunbrack R.L., Thomas G.M. (2023). Palmitoylation-dependent control of JAK1 kinase signaling governs responses to neuropoietic cytokines and survival in DRG neurons. J. Biol. Chem..

[104] Claudinon J., Gonnord P., Beslard E., Marchetti M., Mitchell K., Boularan C., Johannes L., Eid P., Lamaze C. (2009). Palmitoylation of interferon-alpha (IFN-alpha) receptor subunit IFNAR1 is required for the activation of Stat1 and Stat2 by IFN-alpha. J. Biol. Chem..

[105] Zhang M., Zhou L., Xu Y., Yang M., Xu Y., Komaniecki G.P., Kosciuk T., Chen X., Lu X., Zou X. (2020). A STAT3 palmitoylation cycle promotes TH17 differentiation and colitis. Nature.

[106] Wei Y., Li J., Li J., Liu C., Guo X., Liu Z., Zhang L., Bao S., Wu X., Su W. (2024). Dietary long-chain fatty acids promote colitis by regulating palmitoylation of STAT3 through CD36-mediated endocytosis. Cell Death Dis..

[107] Jiang Y., Xu Y., Zhu C., Xu G., Xu L., Rao Z., Zhou L., Jiang P., Malik S., Fang J. (2023). STAT3 palmitoylation initiates a positive feedback loop that promotes the malignancy of hepatocellular carcinoma cells in mice. Sci. Signal..

[108] Ma Y., Liu H., Ou Z., Qi C., Xing R., Wang S., Han Y., Zhao T.J., Chen Y. (2022). DHHC5 facilitates oligodendrocyte development by palmitoylating and activating STAT3. Glia.

[109] Fan X., Zhang S., Sun S., Bi W., Li S., Wang W., Chen X., Fang Z. (2024). GFAP palmitoylcation mediated by ZDHHC23 in spinal astrocytes contributes to the development of neuropathic pain. Reg. Anesth. Pain Med..

[110] Zhao C., Yu H., Fan X., Niu W., Fan J., Sun S., Gong M., Zhao B., Fang Z., Chen X. (2022). GSK3β palmitoylation mediated by ZDHHC4 promotes tumorigenicity of glioblastoma stem cells in temozolomide-resistant glioblastoma through the EZH2-STAT3 axis. Oncogenesis.

[111] Collura K.M., Niu J., Sanders S.S., Montersino A., Holland S.M., Thomas G.M. (2020). The palmitoyl acyltransferases ZDHHC5 and ZDHHC8 are uniquely present in DRG axons and control retrograde signaling via the Gp130/JAK/STAT3 pathway. J. Biol. Chem..

[112] Liu Z.Y., Lan T., Tang F., He Y.Z., Liu J.S., Yang J.Z., Chen X., Wang Z.F., Li Z.Q. (2023). ZDHHC15 promotes glioma malignancy and acts as a novel prognostic biomarker for patients with glioma. BMC Cancer.

[113] Hsiao W.C., Niu G.H., Lo C.F., Wang J.Y., Chi Y.H., Huang W.C., Tung C.W., Sung P.J., Tsou L.K., Zhang M.M. (2023). Marine diterpenoid targets STING palmitoylation in mammalian cells. Commun. Chem..

[114] Matsumoto K., Ni S., Arai H., Toyama T., Saito Y., Suzuki T., Dohmae N., Mukai K., Taguchi T. (2023). A non-nucleotide agonist that binds covalently to cysteine residues of STING. Cell Struct. Funct..

[115] Zhu X.X., Meng X.Y., Zhang A.Y., Zhao C.Y., Chang C., Chen T.X., Huang Y.B., Xu J.P., Fu X., Cai W.W. (2024). Vaccarin alleviates septic cardiomyopathy by potentiating NLRP3 palmitoylation and inactivation. Phytomedicine.

[116] Tsukidate T., Hespen C.W., Hang H.C. (2023). Small molecule modulators of immune pattern recognition receptors. RSC Chem. Biol..

